# Unveiling the Complexities: Exploring Mechanisms of Anthracycline-induced Cardiotoxicity

**DOI:** 10.2174/011573403X322928241021100631

**Published:** 2024-10-31

**Authors:** Rohit Tayal, Ashi Mannan, Shareen Singh, Sonia Dhiman, Thakur Gurjeet Singh

**Affiliations:** 1 Chitkara College of Pharmacy, Chitkara University, Rajpura, Punjab, India

**Keywords:** Cardiotoxicity, oxidative stress, apoptosis, cardiomyocytes, doxorubicin, inflammation

## Abstract

The coexistence of cancer and heart disease, both prominent causes of illness and death, is further exacerbated by the detrimental impact of chemotherapy. Anthracycline-induced cardiotoxicity is an unfortunate side effect of highly effective therapy in treating different types of cancer; it presents a significant challenge for both clinicians and patients due to the considerable risk of cardiotoxicity. Despite significant progress in understanding these mechanisms, challenges persist in identifying effective preventive and therapeutic strategies, rendering it a subject of continued research even after three decades of intensive global investigation. The molecular targets and signaling pathways explored provide insights for developing targeted therapies, emphasizing the need for continued research to bridge the gap between preclinical understanding and clinical applications. This review provides a comprehensive exploration of the intricate mechanisms underlying anthracycline-induced cardiotoxicity, elucidating the interplay of various signaling pathways leading to adverse cellular events, including cardiotoxicity and death. It highlights the extensive involvement of pathways associated with oxidative stress, inflammation, apoptosis, and cellular stress responses, offering insights into potential and unexplored targets for therapeutic intervention in mitigating anthracycline-induced cardiac complications. A comprehensive understanding of the interplay between anthracyclines and these complexes signaling pathways is crucial for developing strategies to prevent or mitigate the associated cardiotoxicity. Further research is needed to outline the specific contributions of these pathways and identify potential therapeutic targets to improve the safety and efficacy of anthracycline-based cancer treatment. Ultimately, advancements in understanding anthracycline-induced cardiotoxicity mechanisms will facilitate the development of more efficacious preventive and treatment approaches, thereby improving outcomes for cancer patients undergoing anthracycline-based chemotherapy.

## 1 INTRODUCTION

With advancements in cancer care leading to better survival rates, cardiovascular diseases have now taken a prominent role as a leading cause of illness and death in cancer survivors [1]. Cancer and Cardiovascular Diseases (CVDs) are the primary reasons for illness and death in developed nations as the mortality linked to cardiovascular diseases is made worse by the application of cancer treatments for patients with cancer. The primary factor leading to drug-related harm to the heart is associated with certain cancer treatments, commonly known as chemotherapy-induced cardiotoxicity [2-4].

In developed nations, the dual challenges of cancer and CVD stand as the foremost causes of illness and mortality. Moreover, the burden of cardiovascular-related deaths is exacerbated by the implementation of cancer treatments in individuals already dealing with cancer, resulting in a major challenge for cardio-oncologists [2, 3]. Progress in cancer treatment methodologies has increased the rates of survival among individuals undergoing chemotherapy, radiotherapy, and hormonal therapy. However, the efficacy of these therapies comes at the cost of notable side effects, manifesting as toxicity in crucial organs, such as the gastrointestinal system, bone marrow, liver, kidney, and cardiovascular tissues [1, 5, 6]. Chemotherapy is the oldest and emerging treatment modality for various cancers, but it can lead to cardiotoxicity, significantly influencing the well-being and prognostic outcomes of individuals afflicted with cancer [7]. Cardiotoxicity refers to structural and functional injury or impairment of the heart that occur during a diseased condition or may be caused by therapy used to treat any illness or disorder [8, 9].

For over three decades, anthracycline antibiotics have been an integral part of clinical practice, gaining widespread acceptance and becoming one of the most successful drug classes utilized in the management of numerous solid carcinomas, breast cancer, and leukemia [2]. Anthracyclines, including doxorubicin (Dox), adriamycin, and epirubicin, are some of the most widely used and potent chemotherapeutic drugs [10]. Their remarkable efficacy and broad applications have solidified their status as a highly utilized and trusted category of drugs [11]. Anthracyclines persist as important components of cancer therapy, often combined with novel targeted drugs of the new generation [1]. However, they have been the most common and main culprit in the induction of cardiotoxicity by chemotherapy. The occurrence of cardiotoxicity caused by anthracyclines is contingent upon the dosage, with a projected cumulative incidence of clinical HF varying between 1% to 5% when the total intake surpasses 400-450 mg/m^2^ [10, 12]. Administration of anthracycline chemotherapy over the course of one year resulted in diminished LVEF in 9% of the patients [13]. When the total dose is 550 mg/m^2^, the cardiotoxicity percentage of anthracyclines ranges from 7% to 16%, while when it is 700 mg/m^2^, the percentage increases to 18% to 48% [14]. The integration of advanced detection technologies, such as cardiac computed tomography, echocardiography, cardiac histopathology, magnetic resonance imaging, and monitoring strategies for cardiac biomarkers, has facilitated early diagnosis and treatment in clinical practice [15]. While clinical assessment can effectively identify high-risk patients, there remains a lack of consensus regarding the optimal strategy for preventing cancer-associated heart disease [16].

Unfortunately, despite extensive research efforts aimed at enhancing clinical approaches for preventing it, there is still no reliable treatment to avoid this complication [17]. Even after nearly five decades of research, anthracycline cardiotoxicity continues to pose a significant challenge in understanding its processes and implementing preventive measures. Hence, it is essential to better explore the molecular mechanisms of these cardiovascular complications and develop more efficient targeted therapy outcomes [17]. This review summarizes the diverse signaling pathways implicated in the pathological processes of anthracycline-mediated cardiotoxicity along with the preclinical and clinically explored therapeutic approaches used in the treatment and prevention. This review also discusses the translational significance of current studies elucidating the complex networks of mechanisms and identifying new targets that can be targeted in the discovery of novel drugs for averting heart disease in cancer survivors.

## 2 ANTHRACYCLINES: STRUCTURE AND CHEMISTRY

Anthracyclines are a significant class of chemotherapy drugs that have been widely used since the 1970s. They were initially extracted from *Streptomyces* spp., which is part of the *Actinobacteria* genus [18]. Due to this, they have played a crucial role in the treatment of numerous cancers. The discovery of anthracyclines, specifically daunorubicin and doxorubicin, was a revolutionary advancement in the field of cancer therapy. The compounds exhibit a wide range of biological diversity that influences their mechanism of action and, consequently, their toxicity [19].

Anthracyclines are composed of a tetracyclic anthraquinone framework, which consists of four rings, and is linked to an amino sugar called daunosamine. This distinctive arrangement enhances their biological functionality [20]. The anthraquinone structure has a quinone-hydroquinone moiety that can undergo redox reactions, which are crucial for its mechanism of action. The process of redox cycling can produce Reactive Oxygen Species (ROS), which play a role in the cytotoxic effects on cancer cells [21]. Daunosamine, an amino sugar, increases the solubility of the molecule and helps it bind with the minor groove of DNA. Anthracyclines intercalate into DNA, causing disruption of crucial cellular processes, like replication and transcription, ultimately resulting in cell death. The intercalative property of the compounds is enhanced by the presence of planar aromatic rings, which insert themselves between the base pairs in the DNA helix [22].

Doxorubicin and daunorubicin are the most clinically relevant anthracyclines, but a large number of derivatives have been designed to improve therapeutic indices or reduce toxicities [23]. The splitting of the anthraquinone core and amino sugar has been utilised to produce analogues that exhibit varying degrees of anticancer efficacy and toxicity [24]. For instance, epirubicin is an epimer of doxorubicin with a modified spatial arrangement of the hydroxyl group on the sugar moiety, resulting in altered pharmacokinetics and a reduced cardiotoxic profile [25]. Similarly, idarubicin, which lacks a methoxy group on the anthraquinone ring, shows increased lipophilicity and better cellular uptake, translating to higher potency against certain leukemias [26]. Liposomal formulations, such as liposomal doxorubicin (Doxil), encapsulate the drug in a lipid bilayer, enhancing its delivery to tumor tissues while sparing healthy cells [27]. These formulations have demonstrated reduced cardiotoxicity and improved patient outcomes in clinical trials. A summary of the types of drugs from anthracycline class has been provided in Table **1**, along with their structure, mechanism of action, indications, and toxicity profile [28-61].

## 3 MOLECULAR MECHANISMS UNDERLYING ANTHRACYCLINE-INDUCED CARDIOTOXICITY

In recent years, the molecular mechanism of anthracycline-induced cardiotoxicity has been extensively studied, including oxidative stress, inflammatory response, mitochondrial dysfunction, autophagy, apoptosis, myocardial fibrosis, Ca^2+^ overload, endoplasmic reticulum stress, and so on. It has been found that anthracycline-induced cardiotoxicity is resulted from a variety of mechanisms, involving multiple signal pathways [62-65]. The development of anthracycline-induced cardiotoxicity has been attributed to a diverse array of mechanisms, encompassing the activation and modulation of multiple signaling pathways within the intricate cellular environment.

### 3.1 PI3k/Akt Pathway

**Fig. (1):**
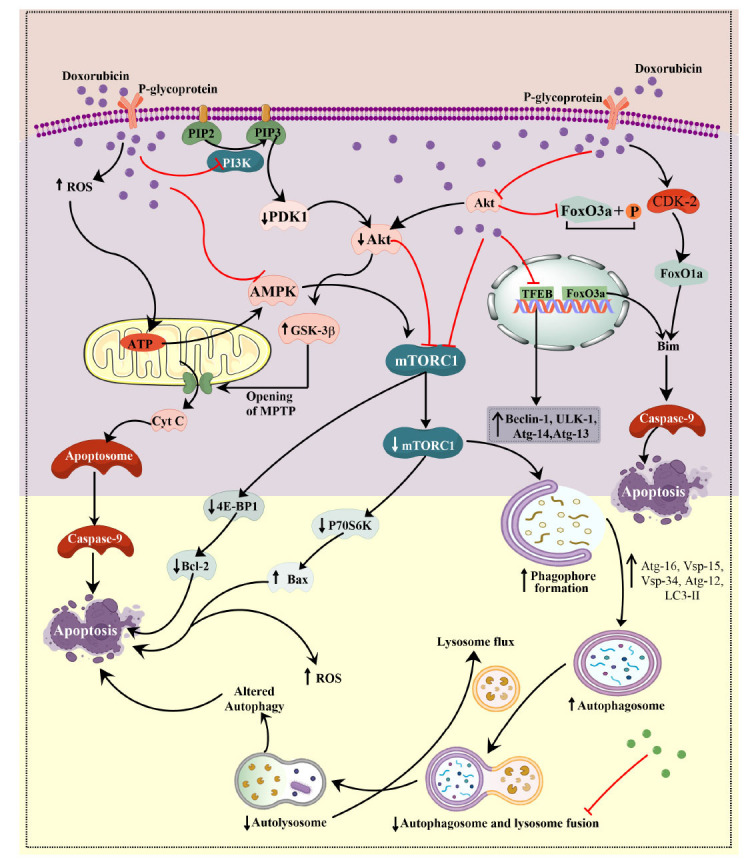
The schematic representation illustrating anthracycline-induced disruptions in the function of key signaling molecules, including PI3K/Akt, GSK-3β, FOXO, mTOR, TFEB, and autophagosome, ultimately leading to an increase in oxidative stress, autophagy, and apoptosis. In particular, Dox inhibits PI3K and Akt activity, which sets off a series of subsequent events marked by the dysregulation of TFEB, FOXO, and mTOR, as well as an increase in GSK-3β activity. This imbalance triggers increased oxidative stress, increased autophagic flux, and amplified apoptotic signaling pathways. Increased activity of GSK-3β promotes the opening of Mitochondrial Permeability Transition Pores (MPTP), which release cytochrome C into the cytoplasm and ultimately cause cell death. Furthermore, Dox directly inhibits AMPK activity, which also attenuates mTOR signaling and disrupts autophagic functions.

The Phosphoinositide 3-kinase/protein kinase B (PI3K/Akt) signaling pathway is a crucial signal transduction pathway that regulates the survival and functioning of cardiac tissue [66-68]. PI3K is an enzyme capable of adding a phosphate group to the hydroxyl group located at the 3-position of the inositol ring found within phosphatidylinositol [69, 70]. Akt, also known as protein kinase B, is a serine/threonine-specific protein kinase that is essential in various physiological activities. Upon activation, Akt modulates a multitude of cellular processes by phosphorylating, activating, or inhibiting a diverse array of proteins, thereby regulating cell division, neovascularization, and survival through the regulation of anti-apoptotic and apoptotic proteins [71-73]. In this pathway, the phosphate group of PIP3 facilitates the binding of PDK1 and Akt protein, recruiting Akt to the plasma membrane and enabling PDK1 to phosphorylate T308, partially activating Akt [74]. The PI3K/Akt pathway assumes crucial functions in the pathogenesis of numerous human disorders, including cardiac hypertrophy, wherein it modulates the cardiomyocyte structure and viability as well as the angiogenic processes [75]. In a mice model of Doxorubicin-induced Cardiotoxicity (DIC), Dox administration resulted in diminished activation of the PI3K/Akt pathway, as indicated by decreased expression of p-Akt protein observed on Western blot analysis. This was concomitant with an elevation in the levels of caspase-3 and Bax, suggesting a potential correlation between reduced PI3K/Akt activity and cardiotoxic effects [76]. In a proteomic study, treatment with Dox and BYL719 (a specific PI3K inhibitor) caused a reduction in the left ventricle mass as well as ejection fraction in mice, resulting in heart atrophy, decreased cardiac output, and myocardial contractility, ultimately leading to right ventricle dilation and cardiac dysfunction [77]. A genomic study showed that administering a constitutively active Akt1 gene to the myocardium *via* adenovirus-mediated intracoronary delivery protected against the development of a DIC model in rats. This study provided strong evidence that activation of Akt signaling can protect against heart damage produced by Dox treatment *in vivo* through increment in LVEF and heart weight [78]. Anthracycline-induced activation of the PI3K/Akt pathway impacts multiple downstream pathways, including mTOR, FOXOs, GSK-3β, Bad, XIAP, Nrf2, NDRG2, and eNOS, regulating the cell cycle and coordinating cardioprotective mechanisms [79] (Fig. **1**). Table **2** includes some further studies on this pathway.

### 3.2 Mammalian Target of Rapamycin (mTOR)

The mTOR is an unconventional serine/threonine kinase found in two distinct complexes. Rapamycin functions by inhibiting the first complex, mTOR complex 1 (mTORC1), comprising mTOR, GβL, DEPTOR, and Raptor. The second complex, mTOR complex 2 (mTORC2), consists of mTOR, GβL, Rictor, Sin1, DEPTOR, and PRR5/Protor-1 [80, 81]. Research has demonstrated that mTOR participates in numerous signaling pathways, such as PI3K/Akt, LKBL/Adenosine 5′-monophosphate-activated Protein Kinase (AMPK), TSC1/TSC2/Rheb, VAM6/Rag GTPases, B-cell lymphoma 1 (Bcl-2), p53 proteins, and Beclin-1 [82, 83]. mTOR exerts its influence on the process of transcription and translation by integrating diverse signaling stimulations, ultimately regulating processes, such as autophagy, apoptosis, and cellular growth [84]. Overall, both mTORC1 and mTORC2 play crucial roles in maintaining cardiac structures, promoting growth and preserving vascular integrity. Furthermore, they are essential for the heart’s ability to adapt to physical stress, facilitating the development of compensatory hypertrophy and mitigating the death of cardiac muscle. During chronic stress, the activation of mTORC1 in the heart has been associated with several detrimental effects, including the promotion of pathological hypertrophy, the onset of energy depletion, and the buildup of improperly folded proteins [85]. Additionally, researchers have demonstrated that suppressing mTORC1 in chronic HF can confer benefits, such as increasing autophagy and decreasing apoptosis, contributing to the prevention of further decline in cardiac function [86, 87]. Moderate suppression of mTORC1, achieved through interventions, such as calorie restriction, rapamycin administration, or utilization of transgenic animal models, has been demonstrated to alleviate cardiac aging [51]. Conversely, in the genetically modified cardiomyocyte-specific mTOR knockout mouse model, it resulted in apoptosis and decreased proliferation, leading to dilated cardiomyopathy with systolic dysfunction [86]. This shows that the balance of mTOR activity in cardiomyocytes is crucial for maintaining optimal cardiac function, as both excessive inhibition and hyperactivation of mTOR can lead to detrimental effects on cardiomyocyte function.

Dox administration has been shown to induce cardiomyocyte apoptosis through the inhibition of AMPK-dependent mTORC1 activity, as indicated by decreased expressions of ribosomal protein S6 kinase (p70S6K) and 4E-binding Protein 1 (4E-BP1) [88]. mTOR-mediated activation of p70S6K serves to suppress apoptosis by mechanisms that include upregulation of anti-apoptotic proteins, like Bcl-2, and deactivation of the proapoptotic protein Bad. Additionally, 4E-BP1 binds to eIF4E when mTOR activity is absent, thereby promoting the translation of proapoptotic proteins and leading to cardiac injury [89]. Furthermore, suppressing AMPK or silencing its activity with siRNA reversed Dox’s effect on mTORC1, emphasizing the significant role of AMPK activation in regulating Dox-mediated mTORC1 inhibition [88]. It was found that Dox suppressed autophagy and contributed to cardiotoxicity by increasing the phosphorylation of p70S6K, a downstream target of mTOR. This suppression of autophagy by Dox activated apoptosis by inducing enzyme caspase 3, which resulted in myocardium death in a rat model [90]. Additionally, the administration of Dox in the mice led to an upregulation of p70S6K protein expression. This upregulation subsequently resulted in the inhibition of autophagy and apoptosis, ultimately leading to the death of cardiomyocytes [91]. While significant research has focused on elucidating the role of mTORC1 in DIC, the role of mTORC2 in this specific scenario is less well-defined [85]. However, mTORC2 controls the phosphorylation and activation of SGK1, which is recognized for its role in promoting the survival of cardiac muscles [92]. Cells exhibiting genetic disruption of mTORC2 demonstrated a lack of SGK1 phosphorylation and an elevated incidence of cell death [85]. In a recent study, it was elucidated that mTORC2 holds a critical role in maintaining contractile performance under acute pressure-overload conditions. Through tamoxifen-induced cardiomyocyte-specific deletion of the Rictor gene in mice, researchers observed the onset of cardiac dysfunction and dilation after just one week of pressure overload, affirming the indispensable contribution of mTORc2 to the heart’s adaptive response to mechanical stress [93]. While considerable attention has been given to understanding the importance of mTORC1 in DIC, the alteration in function of mTORC2 in this context remains less elucidated. Additional preclinical investigations are needed to delineate the precise involvement of mTORC2 in DIC, given that activation of mTORC2 could potentially confer cardioprotective effects.

Moreover, various studies have shown Dox to also initiate autophagy by altering the various pathways that regulate mTOR signaling. Dox was shown to lower the levels of Akt and mTOR by preventing their phosphorylation, which increased autophagy in a rat cardiotoxicity model. On the other hand, sevoflurane, an anaesthetic, improved cardiac damage by upregulating the PI3K/Akt/mTOR pathway and decreasing autophagy [94]. However, Dox enhanced autophagy by triggering its initiation *via* AMPK stimulation and/or mTOR deactivation, promoting phagophore formation and upregulating autophagy-related (Atg) proteins, while also impeding lysosomal proteolysis, leading to the accumulation of autophagosomes and autolysosomes and increased levels of Reactive Oxygen Species (ROS) [95]. Moreover, mTOR-mediated phosphorylation exerts a negative regulatory effect on the nuclear translocation and activity of Transcription Factor EB (TFEB). This downregulation may trigger inflammation in cardiomyocytes upon Dox administration, evident through reduced nuclear TFEB expression alongside heightened phosphorylation of Iĸĸ-αβ and NF-κβ. Dihydrotanshinone I highlights the critical involvement of the mTOR/TFEB/NF-κβ signaling axis in mitigating Dox-mediated inflammation [96, 97]. When cardiomyocytes were treated with dapagliflozin together with Dox, the expression of pro-inflammatory cytokines associated with cardiotoxicity was lowered. Additionally, dapagliflozin inhibited the NLRP3 inflammasome by 27.8% and decreased the levels of mTORC1/FOXO1/3a, directly contributing to the reduction in cardiomyocyte apoptosis [98]. These studies have demonstrated the role of mTOR in the regulation of Dox-induced inflammation by downregulating FOXO and TFEB expression.

Hence, the above evidences demonstrate that the mTOR signaling pathways, influenced by various mediators, including the PI3K/Akt pathway, AMPK, p70S6K, TFEB/ Iĸĸ-αβ/NF-κβ, and FOXO1/3, play pivotal roles in Dox-induced altered autophagy through their inhibition and initiation and contribute to cardiotoxicity (Fig. **1**). In conclusion, the impact of mTOR-regulated autophagy in the context of DIC is twofold. On one hand, the decrease in Dox-induced mTOR downstream signaling within cells, facilitated by AMPK and Akt protein, directly amplifies apoptosis by suppressing p70S6K and 4E-BP1. Conversely, the diminished mTOR signalling induced by anthracycline prompts autophagy through the elevation of autophagy-related proteins, eventually culminating in apoptosis through heightened oxidative stress and inflammation. Table **2** includes some further studies on this pathway.

### 3.3 ULK (Unc-51-like Kinase) Complex

The ULK (Unc-51-like Kinase) complex constitutes a multi-protein assembly, important in initiating autophagy. The primary signaling components initiating the process of autophagy are embodied by the ULK-1 kinase complex [99]. The function of this complex is actively controlled by two prominent signaling pathways, namely mTOR and AMPK. The mTOR is responsible for inhibition, while AMPK is responsible for activating ULK-1 [100]. The mTORC1 serves as a central hub for regulating autophagy signaling. It interacts with the ULK-1 complex, inhibiting the Beclin-1 phosphorylation by ULK-1, which is necessary for initiating autophagy. This interaction involves the formation of a complex comprising Beclin-1, Atg16L, Vps34, Vps15, Light Chain kinase (LC) I and II, and p62 [100, 101].

Although, it is very difficult to conclude the role of ULK-1-mediated autophagy in cardiomyocyte growth and pathological conditions. Inducible cardiomyocyte-specific ULK-1 knockout mice exhibited increased autophagic activity, reduced ejection fraction, and enlargement of the left ventricle. This suggests that the absence of ULK-1 in adults, but not ULK-2, rapidly leads to fatal dilated cardiomyopathy with significant cellular and molecular irregularities in the heart muscle [102]. Deletion of ULK-1 and ULK-2 enhanced autophagy in cardiomyocytes, resulting in age-related HF. There is growing evidence showing that Dox elicits cellular alterations, which are associated with the onset of autophagy and the formation of autophagosomes in cardiac cells. Exposure to Dox inhibited the mTOR signaling pathway, promoting Beclin-1 phosphorylation through ULK-1 activation [103, 104]. Dox enhanced autophagy by increasing Atg5 and Atg12 proteins, but resveratrol countered Dox-induced autophagic action to enhance the survival of heart cells [105]. Dox reduced mTOR signaling and activated Beclin-1 phosphorylation through ULK-1, leading to the induction of autophagy. Elevated AMPK phosphorylation exacerbated this process by activating the Beclin-1 and ULK-1 complex, subsequently initiating Vps34 and Vps15 activation, facilitating autophagosome formation and phagosome extension through the recruitment of additional Atg proteins [106]. Despite this, when evaluating the subsequent process of autophagy, Dox hindered the flow of autophagy and suppressed the acidification of lysosomes in cardiomyocytes. The interruption in the autophagic process caused a buildup of undegraded autolysosomes, subsequently leading to cardiotoxicity by generating ROS [107]. The reported findings offer crucial evidence regarding the cellular alterations and onset of autophagy in cardiac muscles following exposure to Dox.

On the other hand, TFEB undergoes phosphorylation by both mTORC1 and Akt independently, leading to its cytoplasmic retention. Under stress, like nutrient scarcity, TFEB undergoes dephosphorylation, relocating to the nucleus to regulate target gene expression and activate autophagy [108]. Interestingly, anthracycline therapy caused TFEB depletion, leading to the suppression of proteins involved in macro-autophagy (ULK-1, p62, and LC3) and chaperone-mediated autophagy (LAMP-2A and hsp90), resulting in impaired lysosome activity autophagy flux [109]. In an *in vitro* study, the presence of Dox was found to inhibit critical processes in the canonical autophagy pathways, including LC3 cleavage, ULK1 phosphorylation, and SQSTM1 degradation, and lead to the promotion of apoptosis and inflammation. When the sesqui-neolignan compound isodunnianol was administered, it reversed these effects by stimulating autophagy *via* upregulation of the AMPK/ULK-1 pathway independently of mTOR [110]. The early disruption of autophagosome formation, prolonged elevation of autophagy-associated proteins, and resulting effects on lysosomal activity highlight the complex relationship between Dox and the regulatory network governing autophagy in cardiomyocytes.

### 3.4 Forkhead Box O (FOXO)

The FOXO proteins are essential for controlling various bodily functions, including cell cycle control, responding to oxidative stress, facilitating autophagy, regulating protein degradation, and aiding in recovery processes [111-113]. The genes activated by FOXO in these processes encompass cyclin-dependent kinase inhibitor 1B (p27, KIP1), GADD45, Atg, MnSOD, DDB1, FasL, and catalase [114]. FOXO exhibits its four isoforms (FOXO1, FOXO3, FOXO4, and FOXO6) with FOXO3 being the predominant isoform expressed in the heart. FOXOs are modulated by various upstream molecules, including, AMPK, ERK, c-Jun N-terminal kinase (JNK), Akt, and SIRT1/3 [115]. FOXO3 has been demonstrated to suppress or reverse myocardial hypertrophy in numerous preclinical studies [116, 117]. Inhibition of FOXO3 caused abnormal enlargement of heart muscle cells, ultimately resulting in HF and mortality [116, 117]. The expression of FOXO3a led to an elevation in oxidative stress, inflammatory markers, and apoptosis [118]. Dox exposure triggered CDK-2-mediated FOXO1 activation, leading to Bim transcription and subsequent cardiomyocyte apoptosis and atrophy, with the protection afforded by FOXO1 inhibition or siRNA-mediated knockdown in cultured cells [119]. Recent studies have revealed the cytostatic and cytotoxic properties of Dox [120, 121], facilitated by FOXO activation, predominantly through the downregulation of the PI3K/Akt pathway [122]. *In vitro* findings have demonstrated the overexpression of the FOXO3a protein to alleviate mitochondrial fission and apoptosis in response to Dox. Moreover, transgenic mice overexpressing FOXO3a protein, specifically in the heart, exhibited resistance to Dox-induced damage by inhibiting transcription of the mitochondrial dynamic protein of 49 kDa [123]. NaHS treatment prior to Dox exposure in H9c2 cells surpassed p- FOXO3a and p-Akt, attenuating FOXO3A nuclear translocation and inhibiting Dox-induced apoptosis [124]. In the heart, SIRT1 increased Bcl-xL expression and inhibited proapoptotic factors, like Bax, and activated caspase 3 by stimulating FOXO [125]. SIRT3 activated FOXO3a, enhancing the transcription of SOD by deacetylation in response to oxidative stress [126]. FOXO1 and FOXO3a were deacetylated by SIRT3, improving their activity and inhibiting apoptosis [127]. Daidzein, an isoflavone, exhibited a cardio-protective effect in the DIC model by decreasing cardiac inflammation, apoptosis, and fibrosis, and by activating the SIRT3/FOXO3a cascade [128] (Fig. **1**). Table **2** includes some further studies on this pathway.

### 3.5 Glycogen Synthase Kinase (GSK-3)

The GSK-3 family includes GSK-3α and GSK-3β, first identified to be involved in insulin signaling. These types of GSK-3 are always active inside cells and work as enzymes that help modify proteins. GSK-3 activity is enhanced through phosphorylation at tyrosine residues (Thr 279 for GSK-3α and Thr 216 for GSK-3β) [129-131] and attenuated *via* serine phosphorylation (Ser 21 for GSK-3α and Ser 9 for GSK-3β) [132]. The activation of Akt leads to the phosphorylation of GSK-3β, a kinase active in quiescent cells that undergoes inactivation through phosphorylation [133].

However, the knockdown of GSK-3α and GSK-3β in cardiomyocytes caused HF in mice, as evidenced by DNA damage, apoptotic cell death, and disrupted mitochondrial morphology and nuclear enlargement in histopathological examination [134]. However, the role of Ser 21 and Ser 9 has been studied individually in cardiac pathological state; the prevention of cardiac decompensation under pressure overload was seen when the S9 of GSK-3β was maintained in an unphosphorylated state. The phosphorylation of S21 of GSK-3α prevented the compensatory increase in cellular proliferation in the cardiac tissue [135]. A study has demonstrated the targeted removal of GSK-3β to be linked with a notable augmentation in the proliferation of cardiomyocytes. This effect is further amplified when the stressors of either transverse aortic constriction or myocardial infarction are introduced [136]. It has been concluded that GSK-3 inhibitors might potentially provide both positive and negative outcomes in cardiovascular illnesses. Therefore, directing efforts toward targeting the particular isoform of GSK-3 may present more efficacious therapeutic strategies for the prevention of cardiac diseases. A recent investigation revealed that Dox lowered GSK-3β expression in the cardiotoxicity model of rats [137]. Another study demonstrated that adriamycin remarkably reduced the protein levels of p-GSK-3β, p-PI3K, and p-Akt in rat heart muscle and increased apoptosis, leading to myocardial damage [138]. Furthermore, Dox suppressed the phosphorylation of GSK-3β and Akt protein, resulting in elevated cell death and stress levels in the heart tissue [139]. Moreover, GSK-3α regulated mPTP opening during HF, resulting in Bax overexpression and apoptosis in cardiomyocytes [140], while GSK-3β delayed mPTP opening and provided cardioprotection [141]. Dox reduced the levels of p- GSK-3β, resulting in the opening of mPTP and apoptosis, which could be reversed by co-administration of cryptotanshinone, suggesting a protective mechanism against DIC *via* Akt/GSK-3β/mPTP pathway [142]. Morphine preconditioning demonstrated to be cardioprotective by reducing the infarct size in the DIC rat model *via* activation of GSK-3β by ERK, independently of PI3K [143]. Dox induced pyroptosis through enhanced inflammation and death in cardiac tissue *via* suppression of the GSK-3β/caspase-1/GSDMD pathway [144]. GSK-3β has also been associated with the modulation of an assembly of transcription elements, including β-catenin, NF-κβ, AP-1, NF-AT, and CREB [133]. The lack of research on these linkages and GSK-3α in the framework of AIC makes it evident that more *in vivo* investigations are necessary to fully understand these pathways (Fig. **1**). Table **2** includes some further studies on this pathway [145-160].

### 3.6 MAPK (Mitogen-activated Protein Kinase)/ERK (Extracellular Signal-regulated Kinase) Pathway

**Fig. (2):**
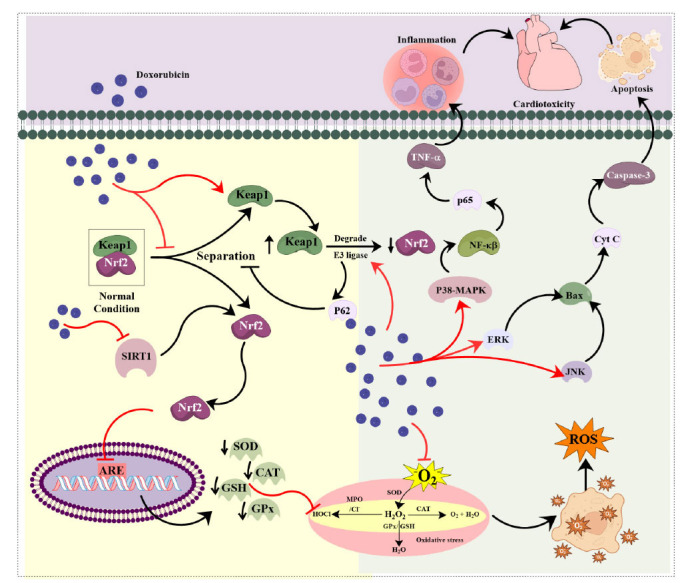
The schematic depiction showing the mechanism underlying DIC, focusing on its impact on the Nrf2/Keap1/ARE and MAPK/ERK signaling pathways. Under normal conditions, Nrf2 and Keap1 are typically bound together, but separation occurs in response to stress stimuli. Dox impedes this separation process, leading to heightened oxidative stress as Nrf2 fails to activate the synthesis of ARE genes, including those encoding SOD, CAT, GSH, and GPx. This elevated oxidative stress activates the p38-MAPK and ERK pathways, resulting in the upregulation of NF-κβ and Bax proteins. Consequently, this cascade of events induces inflammation and apoptosis in cardiomyocytes, thereby contributing to cardiotoxicity.

MAPKs are a class of protein kinases that control essential cellular functions, like growth, response to stress conditions, programmed cell death, and defense against infection by undergoing a cascade of autophosphorylation and phosphorylating substrates [161-163]. The well-known MAPK pathways, namely ERK1/2, JNK, ERK5, and p38, have been linked to several facets of cardiac mechanisms from the development process to pathological alterations [164]. MAPK/ERK pathway comprises a small G protein (RAS) and three protein kinases (RAF, ERK, MEK), which are activated upon ligand binding to an RTK, leading to a signaling cascade culminating in ERK nuclear translocation and stimulation of transcription components that control the gene expression [76]. ERKs are activated through several factors, including cytokines and growth factors, which trigger signaling cascades involving molecules, including Ras, PKC, tyrosine kinases, Ca^2+^, PKA, and the enzymes MAPK/ERK kinase (MEK) and Raf-1 [165-168]. The MAPK family is significantly implicated in apoptosis in cardiomyocytes, as daunomycin triggers the activation of MAPKs through ROS and Ca^2+^. Specifically, ERKs play a protective role against cardiomyocyte apoptosis, while p38 MAPK is implicated in inducing apoptosis in cardiomyocytes [169]. Daunomycin induced the dose-dependent upregulation of three members of the MAPK family in cardiac myocytes, such as JNK, ERKs, and p398MAPK. Oxyradical scavengers or Ca^2+^ chelators blocked the activation of ERKs and p38MAPK caused by daunomycin [169]. *In vitro* research findings suggest a functional association between ERK1/2 and p53, indicating that the ERK/p53 cascade serves as the upstream signaling pathway accountable for Dox-triggered cardiomyocyte death. The significance of ERK1/2 and p53 in the DIC was underscored by utilizing the p53 inhibitor pifithrin-α and the ERK inhibitor U-0126, which effectively reversed the apoptotic cell death induced by Dox [170]. Moreover, exposure to Dox led to increased levels of phosphorylation of the NF-ĸβ p65 and phosphorylated p38 MAPK subunit, a response that was significantly attenuated by SB203580, a selective inhibitor of p38 MAPK [171]. MAPK activation triggered the subsequent upregulation of transcription factors, such as NF-ĸβ, serving as a critical mediator for the transcriptional control of numerous genes implicated in immune system functionality as well as inflammation and acute response pathways [172]. The immune response stands as a key function controlled by MAPKs, triggering the synthesis of immunomodulatory cytokines, such as IL-1, IL-12, TNF-α, and IL-10, upon upregulation of the ERK, p38MAPK, and JNK pathways [173, 174]. Several substances, including pristimerin and diallyl trisulfide, have demonstrated the mitigation of DIC by reducing inflammatory markers through inhibition of the MAPK/NF-ĸβ pathway [175, 176]. Hence, suppression of the MAPK pathway might offer therapeutic advantages in managing anthracycline-induced cardiotoxicity, although the role of ERK5 remains unexplored in pre-clinical settings (Fig. **2**). Table **3** includes some further studies on this pathway.

### 3.7 Nrf2/Keap1 Signaling Pathway

In physiological conditions, Nuclear factor erythroid 2-related factor 2 (Nrf2) functions as a transcription factor, modulating the expression of antioxidant genes. However, it remains inactive and does not induce downstream gene expression [130, 177]. Nrf2 predominantly exists within cells in a complex formed by its interaction with Keap1 [178]. Kelch-like ECH-associated protein 1 (Keap1) facilitates the ubiquitination and subsequent cytoplasmic degradation of Nrf2 through the activity of E3 ubiquitin ligase comprising cullin-3. The absence of Keap1 results in increased Nrf2 activity, leading to increased expression of downstream antioxidant genes [179]. Under oxidative stress, Nrf2 activation involves dissociation from the Nrf2-Keap1 complex, leading to nuclear translocation and binding to Antioxidant Response Elements (AREs) in gene promoter regions. This results in the increased expression of diverse antioxidant enzymes, including CAT, SOD, and GPx, which aid in the mitigation of ROS [178, 180]. An *in vitro* investigation identified Nrf2/Keap1 pathway to serve as a key early response to acute oxidative damage generated by Dox. The primary regulating actions involved oxidation and autophagic breakdown of the antioxidant sensor Keap1 protein [181]. Dox possesses the capability to impede the dissociation of Keap1 from Nrf2, consequently constraining the nuclear translocation of Nrf2, thereby hampering the manifestation of its subsequent anti-ferroptosis elements, including HO^-1^ and GPx. This, in turn, facilitates a reduction in glutathione synthesis, which results in the buildup of lipid peroxides [182, 183]. The suppression of Nrf2 expression resulted in heightened intracellular oxidative stress, augmented levels of apoptosis, and diminished cellular viability [184]. Dox administration has been reported to have the potential to elevate Keap1 levels and suppress Nrf2 and NAD(P)H oxidase expressions, thereby exacerbating stress conditions in the cell [185]. Moreover, Sirt1 is recognized as a crucial controller implicated in inflammation, cell death, and the body's defense against harmful oxidants. Studies have shown that Sirt1 triggers the Nrf2 pathway, which mitigates oxidative stress [186]. A study demonstrated that cardiac-specific Sirt1^−/−^ knockout mice reflected elevated oxidative stress and ferroptosis when exposed to Dox. Dox reduced the movement of Nrf2 to the nucleus and a lack of Sirt1 additionally hindered this process, along with the subsequent Keap1 pathways, leading to heart damage. This study concluded Sirt1 to have a protective function in reducing DIC by regulating ferroptosis through the Nrf2/Keap1 pathway [187]. Furthermore, DIC could be prevented through activation of Nrf2/Sirt2 signaling in cardiomyocytes by increasing the levels of ARE and reducing ROS levels [188]. After exposure to Dox, an increase in the activity of Tripartite Motif containing-21 (TRIM21) has been observed, which subsequently impaired the functioning of Nrf2. TRIM21, acting as an E3 ubiquitin ligase, associated with p62 to disrupt the dissociation of Nrf2 from Nrf2-keap1, consequently impeding Nrf2 function and subsequently reducing the expression of antioxidant genes downstream. Blocking or reducing TRIM21 activity may boost Nrf2 levels and reduce the severity of DIC [189]. A preclinical investigation found that microRNA-200a (miR-200a) can act on Keap1 mRNA, leading to the degradation of Keap1 mRNA. This process could activate Nrf2 and effectively shielded mice from DIC [190]. Another study explained that reducing miR-152 led to increased inflammation, oxidative stress, and the death of heart muscle cells when exposed to Dox [191]. Furthermore, PKC, P13K/Akt, and the p21 protein exhibited the ability to activate Nrf2 through interaction with Keap1, leading to its phosphorylation (Fig. **2**) [192]. However, the crosstalk between these regulating proteins is not well-defined, which may be potential target for future research. Table **3** includes some further studies on this pathway [193-213].

### 3.8 NOTCH (Neurogenic Locus Notch Homolog Protein) Signaling Pathway

**Fig. (3):**
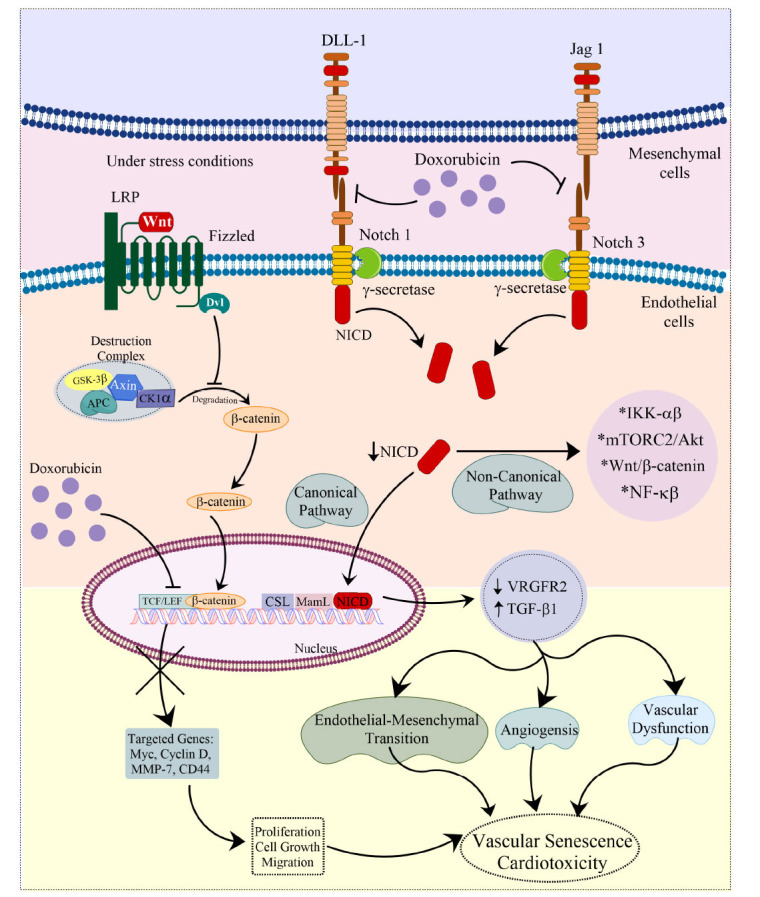
The depicted figure illustrates the inhibitory effect of Dox on the binding of NOTCH receptor to DLL-1 and Jag-1 ligands on sender cells, thereby resulting in decreased VEGFR levels and increased TGF-β1 expression in endothelial cells. This inhibition contributes to vascular senescence and cardiotoxicity. Additionally, Dox suppresses the transcription of β-catenin by the TCF/LEF transcription factor under stressful conditions, leading to diminished proliferation and migration of endothelial cells.

The NOTCH signaling pathway involves a group of transmembrane receptors called NOTCH receptors (NOTCH1, NOTCH2, NOTCH3, NOTCH4) and their corresponding ligands (*e.g.*, Delta-like ligands, DLL1, DLL3, DLL4, Jagged 1, and Jagged2). These receptors and ligands are cell surface receptors, initiated by direct cell-cell communication [214, 215]. When a signaling molecular (NOTCH ligand) from one cell is attached to a specific protein (NOTCH receptor) on another cell, it initiates a sequence of proteolytic processes. The NOTCH receptor undergoes cleavage by proteases, including gamma-secretase, resulting in the liberation of the intracellular domain of NOTCH (NICD) [70, 216]. The NICD is released into the cytoplasm and subsequently translocated to the cell nucleus. In canonical signaling, it interacts with CSL in the nucleus, leading to the recruitment of MAMLs. This action results in the release of corepressors and the recruitment of coactivators, ultimately enhancing the transcription of genes targeted by NOTCH [217]. The activation of NOTCH target genes leads to an alteration in the expression of genes, which can have diverse downstream effects [218]. In the non-canonical signaling pathways, NOTCH interacts with Wnt/β-catenin, mTORC2/Akt, NF-κβ, Iĸĸ-α/β, and PINK1 on mitochondria without relying on CSL [219, 220]. Research has shown that active NOTCH1 is predominantly present in endocardial cells located beneath the trabecular membrane and is responsible for pro-survival stimulus in the cardiac and endothelial tissues [219, 221]. The NOTCH signaling pathway influences the levels of cadherin 5 and BMP2, belonging to the Transforming Growth Factor-β (TGF-β) family. Furthermore, the NOTCH signaling pathway promotes Epithelial-mesenchymal Transition (EMT) in atrioventricular canals by reducing the activity of Vascular Endothelial Growth Factor Receptor 2 (VEGFR2), an important inhibitor of EMT [222, 223]. A recent investigation found that mesenchymal stem cells prevented H9c2 cells from Dox-induced vascular aging by releasing VEGF, which triggered the Jagged/Notch-1 signaling pathway, consequently suppressing the release of TGF-β1 [224]. Dox leads to an enlarged heart condition that varies with dosage when administered to cancer patients. This effect is linked to the hindrance of endothelial cell growth, movement, and blood vessel formation by TGF-β1 [225]. Another study revealed that blocking the NOTCH pathway hindered the process of rebuilding both the structure and functions of the heart after exposure to Dox in adult zebrafish. The findings showed that damage to the heart caused by Dox led to changes in the structure of the ventricles, followed by stimulation of the NOTCH pathway, which encourages the heart to regenerate and regain its ability to contract effectively (Fig. **3**) [226]. Adriamycin caused harmful changes in heart cells by triggering cell death, enlargement, and excessive tissue formation through the suppression of NOTCH1 in H9c2 cells [227]. The role of specific canonical and non-canonical NOTCH pathways has not been explored to much extent, so further research is needed to establish a proper intervention to target this pathway.

### 3.9 Wnt/β-catenin Signaling

The Wnt pathway, also known as the Wnt/β-catenin signaling pathway, is an essential cellular communication route that contributes to development processes, maintaining tissue stability and influencing disease conditions. This pathway is initiated by extracellular signaling molecules called Wnt ligands, which are secreted glycoproteins that attach to cell surface receptors called Frizzled (FZD) receptors [228, 229]. When a Wnt molecule attaches to the FZD receptor alongside its co-receptor LRP5/6, it initiates a cascade of internal processes that lead to the stimulation of the Wnt pathway [230]. This process can vary depending on whether the Wnt pathway is categorized as canonical (β-catenin-dependent) or non-canonical (β-catenin-independent). In the canonical pathway, Wnt signaling leads to the cytoplasmic stabilization and accumulation of β-catenin. Normally, when Wnt signaling is not present, a “destruction complex” consisting of proteins, such as GSK-3β, CK1, and APC, phosphorylates β-catenin, directing it towards proteasomal breakdown [231, 232]. Active Wnt signaling inhibits the destruction complex, enabling the cytoplasmic accumulation of β-catenin. Then β-catenin moves to the nucleus, where it attaches to the TCF/LEF transcription factors and activates specific target gene transcription, influencing cell fate, proliferation, and differentiation [233, 234]. Non-canonical Wnt signaling promotes vascular oxidative stress by upregulation of NADPH oxidase, endothelial dysfunction, and inflammation, and is responsible for phenotypic changes in the smooth muscle cells, resulting in fibrosis and myocardial dysfunction [235]. Furthermore, Dox decreased TCF transcriptional response dependent on the β-catenin in the H9c2 cells, which was reversed by the addition of Yangxin [236]. A genomic study revealed that Dickkopf-1 (Dkk-1) levels increased in the DIC and exacerbated H9c2 cells death by apoptosis and mitochondrial dysfunctioning. Furthermore, the upregulation of the Wnt/β-catenin pathway counteracted the impact of elevated Dkk1 levels and mitigated Dox-induced cardiotoxicity [237]. A proteomic study showed that PKC-ζ phosphorylation increased after exposure to Dox and overexpression of PKC-ζ worsened the cardiac damage. The use of siRNA to reduce PKC-ζ levels alleviated the heart damage caused by Dox, while the activation of the Wnt/β-catenin pathway prevented the worsening of Dox-induced heart damage by counteracting the overexpression of PKC-ζ [238].

Long intergenic non-coding RNA-p21 (lincRNA-21) levels were found to be significantly elevated in HL-1 murine cardiomyocytes and the expression of β-catenin to be suppressed after Dox treatment. In addition, Dox treatment led to a significant increase in the expressions of senescence-related genes p53 and p16, whereas telomere size and the activity of telomerase were reduced. This study suggested that suppressing lncRNA-p21 could prevent Dox-induced heart damage by controlling the Wnt/β-catenin pathway [239]. Additionally, the role of the DDX3X protein of the RNA helicase family and regulator of Wnt/β-catenin signaling has been evaluated in the DIC. This study showed the Wnt/β-catenin signaling and DDX3X protein expression to be downregulated in H9c2 cardiomyocytes when exposed to Dox. Furthermore, DDX3X knockdown worsened heart muscle cell death and dysfunction in mitochondria caused by Dox treatment, while increasing the levels of this protein reversed cell death by activating the Wnt/β-catenin signaling pathway (Fig. **3**) [240]. Further pre-clinical research is essential to elucidate the precise mechanisms underlying this innovative approach for both the treatment and prevention of anthracycline-induced cardiotoxicity. Table **4** includes some further studies on this pathway [236, 241, 242].

### 3.10 Toll-like Receptor (TLR) Signaling

**Fig. (4):**
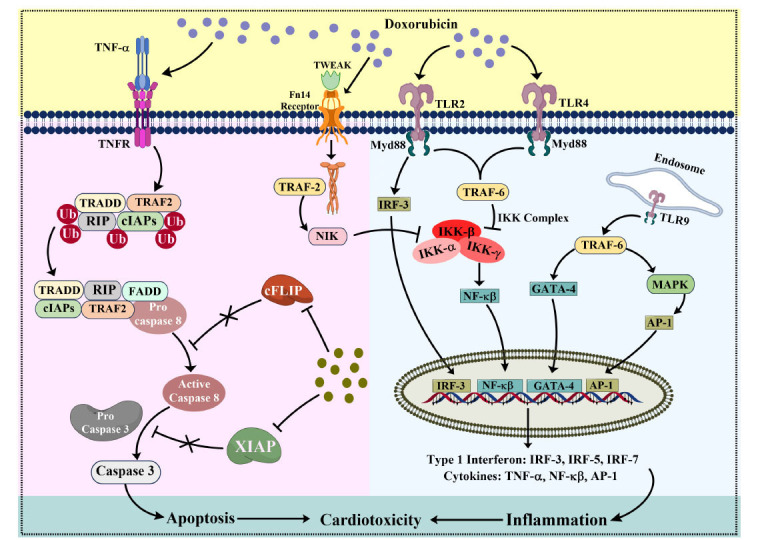
The direct stimulation of TLR by Dox, leading to suppression of the Iĸĸ complex and the subsequent activation of NF-κβ, AP-1, and GATA. The activation of these proteins further stimulates the synthesis of interferons and cytokines, leading to an inflammatory response. Additionally, Dox activates Tweak binding with the Fn14 receptor, promoting inflammation through the upregulation of TRAF-2 and NIK components. The increase in cytokine levels activates the TNF Receptor (TNFR), initiating apoptosis *via* the extrinsic pathway. Furthermore, Dox inhibits cFLIP and XIAP, exacerbating apoptosis in cardiomyocytes and contributing to cardiotoxicity.

TLRs are a class of Pattern Recognition Receptors (PRRs) that are essential for triggering the innate immune response [243]. TLRs are found in different cellular compartments, including the intercellular compartments, such as endosomes (*e.g.* TLR3, TLR7, TLR8, TLR9) and cell surface (*e.g.* TLR1, TLR2, TLR4, TLR5, TLR6) [244]. These markers are present in all inherent immune cells, including basophils, macrophages, natural killer cells, neutrophils, dendritic cells, eosinophils, and mast cells [245]. TLRs, when activated by DAMPs, recruit adapter proteins and trigger downstream signaling cascades involving kinases. As a result, pro-inflammatory transcription factors (NF-κβ, IRF3, AP-1) are transported into the nucleus, where these regulate the activation of genes responsible for producing immune-related substances, like interferons, cytokines, chemokines, and antimicrobial peptides [246, 247]. TLRs generate a rapid reaction to both harmful and harmless molecules released by injured tissues. Among the classes that have been examined thus far, it has been observed that TLR2, TLR4, TLR3, TLR5, TLR7/8, and TLR9 are mainly involved in the pathophysiology of the heart [248]. DIC is characterized by increased oxidative stress associated with the upregulation of TLR2, which initiates NF-κβ and ultimately results in apoptosis [249, 250]. Moreover, TLR2 knockout mice exhibited reduced activation of NF-κβ, decreased release of pro-inflammatory cytokines, and less caspase-3 activation after Dox treatment [251]. The stimulation of TLR4 by Dox may arise from oxidative stress, leading to myocardial cell injury through the promotion of lipid peroxidation in the cellular membrane [244]. The activation of TLR4 during the administration of Dox resulted in oxidative stress, apoptosis, and inflammation of the heart, while also leading to increased levels of endothelial-1, contributing to dysfunction in the left ventricle. The TLR4 knockout lowered the generation of ROS in the cardiac tissue and inhibited the decrease of the transcription factor GATA-4, thereby potentially reducing the DIC [252]. In an *in vitro* study, the administration of Dox to H9c2 cardiomyocytes resulted in elevated levels of TLR4 expression and subsequent activation of signaling pathways, including MyD88, NF-κβ, IL-1 β, IL-6, and TNF-α, alongside a reduction in Iĸĸ-β expression [253]. However, *in vivo* studies have shown DIC to lead to an increase in the activity of the TLR4 signaling pathway, resulting in inflammation, changes in heart structure, oxidative stress, cell death, and reduced heart function [250]. However, TLR5 deficiency lessened the severity of DIC and enhanced heart function. The process by which TLR5 contributes to heart injury involves the activation of NOX2 and the production of superoxide, triggered by the direct contact of TLR5 with SyK, followed by p38 activation [254]. Additionally, the absence of TLR9 inhibited the p38 MAPK upregulation while Dox was administered, potentially facilitating autophagy through the TLR9-p38 MAPK signaling pathway. The TLR9 deletion provides protection against heart damage by Dox through the promotion of p38-dependent autophagy (Fig. **4**) [255]. Most of the studies have explored the role of TLR2 and TLR4 in anthracycline-induced cardiotoxicity; thus, the significance of other TLRs, such as TLR3, TLR5, TLR6, TLR7/8 and TLR9, still needs to be investigated. Table **5** includes some further studies on this pathway.

### 3.11 Nuclear Factor-kappa β (NF-κβ) Pathway

The NF-κβ pathway plays a vital role in controlling immunological reactions and inflammation through signaling cascades. It consists of several transcription factors that have a key function in regulating the activation of genes linked to inflammation, immunity, cell survival, and various physiological processes [235, 256, 257]. The NF-κβ pathway is stimulated by various stimuli, such as infections, cellular stressors, and cytokines. The NF-κβ family comprises many transcription factors, predominantly the p50 subunits and p65 (RelA). Normally, NF-κβ is located in the cytoplasm and associated with inhibitory proteins called Iκβs (Inhibitor of κβ) [258]. Iκβs trap NF-κβ in the cytoplasm, inhibiting its nuclear entry and gene expression activation. The NF-κβ stimulation results in the production of pro-inflammatory cytokines, which attract immune cells to the location of infection or tissue injury [259]. The inhibition of this activation significantly reduced ventricular enlargement and structural changes in heart muscle caused by TNF-α in cardiomyopathy [260]. The administration of Dox stimulated NF-κβ activation within cardiac cells, leading to an inflammatory reaction marked by the discharge of pro-inflammatory cytokines, such as IL-1β and TNF-α, within the cardiac tissue [261, 262]. Additionally, the presence of Dox in rats caused a rise in ROS, resulting in increased levels of NF-κβ in the cardiac tissue [263]. Dox increased NF-κβ protein levels and inhibited Iκβs phosphorylation in rats, worsening heart toxicity [264]. Exposure to Dox resulted in increased levels of phosphorylated p38 MAPK and phosphorylation of the NF-κβ p65 subunit in H9c2 cardiac cells. The research indicates that Dox exposure led to decreased cell viability and triggered an inflammatory reaction, as evidenced by elevated levels of IL-1β, IL-6, and TNF-α [171]. NF-κβ was further activated in cardiomyocytes by various other pathways or proteins in the context of DIC. For instance, Dox upregulated the gene expressions of Rac1 and Fn14, resulting in the elevation of p53, NF-κβ, p65, and INF-γ levels. Hence, it can be concluded that Dox induces cardiotoxicity and inflammation by activation of Rac1/TWEAK/Fn14/NF-κβ pathways [260] (Fig. **4**). Table **5** includes some further studies on these pathways [261-281].

### 3.12 Interferon Regulatory Factor (IRF) Pathway

The IRF pathway is a series of signals responsible for controlling the production of interferons and the immunological reaction to viral infections and other harmful agents. Interferons are signaling proteins essential for defending against viruses and triggering adaptive immune and innate responses [282]. The activation of IRF3 and IRF7 occurs when infected cells recognize viral nucleic acids using PRRs, like TLRs and RLRs, in the initiation pathway. Phosphorylation by several kinases, such as TBK1 and Iĸĸ, is essential for the activation of IRF3 and IRF7 [283]. After phosphorylation of IRF3 and IRF7, they attach together to form dimers and relocate to the nucleus of the cells where they function as transcription factors, stimulating the activation of various genes, including those responsible for type I interferons (IFN-α and IFN-β) and immune-related proteins, like IL-6, TNF-α, and chemokines, such as CXCL10, through their interaction with specific DNA sequences [283]. The immunological response to a cardiac injury, such as myocardial infarction or HF, may involve induction of the IRF pathway. While this response is essential for tissue repair, excessive inflammation can lead to adverse effects on the heart's structure and function [284]. The process involving cyclic GMP-AMP synthase (cGAS) and Stimulator of Interferon Genes (STING) is a pathway used to trigger the body’s natural immune response. The endoplasmic reticulum-associated STING pathway could potentially trigger both NF-κβ and IRF3 transcriptional pathways, leading to increased production of type I interferon. Several research studies have demonstrated that the cGAS/STING pathway is involved in both inflammation and immune response across a range of diseases [285]. A research study analyzed the impact of DIC in mice by examining the cGAS/STING pathway and IRF3 expression through proteomic methods. The study demonstrated significant activation of the cGAS/STING pathway in cardiac endothelial cells, resulting in increased CD38 expression mediated by IRF3, which subsequently lowered NAD levels and led to mitochondrial dysfunction through CD38 intercellular NAD glycohydrolase (NADase) activity [286]. The suppression of STING resulted in improved survival rates and cardiac function as well as reduced levels of inflammatory cytokines in the cardiomyocytes. This study demonstrated that blocking STING could potentially lessen the harmful effects of DIC in mice [285]. Furthermore, mice with knockdown of cGAS and IRF3 protein significantly attenuated cardiotoxicity, having an established critical role in pathology [286].

A recent investigation found that the use of Dox enhanced the discharge of IFN-γ in heart muscle cells [287]. In cardiomyocytes treated with Dox, IFN-γ inhibited the AMPK/Acetyl-CoA Carboxylase (ACC) pathway *via* p38-related mechanism, leading to disturbances in the restructuring of fatty acid metabolism and a decrease in mitochondrial respiratory capacity [285]. In another study on cardiotoxicity in rats, Dox increased inflammatory IFN-γ levels and caused overexpression of NF-κβ and TNF-α, and as well as prevented curcumin administration [276]. A recent study showed that temporarily inhibiting IFN-γ improved heart function damaged by Dox without negatively impacting tumor growth or spread of cancer [288]. Moreover, it has been noted that IFN-γ disrupted AMPK signaling by suppressing the AMPK/ACC axis through a pathway dependent on p38, thereby increasing the cardiotoxic effects of Dox [287]. The antibody therapy directed at IFN-γ not only enhanced heart performance in mice, but also showed that blocking IFN-γ could relieve both new and old DIC, without compromising Dox’s effectiveness against tumors in mice [288]. IRF-mediated signaling could be explored as a potential therapeutic approach to mitigate the inflammatory and immune responses associated with cardiotoxicity.

### 3.13 cFLIP (Cellular FLICE-inhibitory Protein)

cFLIP is a regulatory molecule responsible for hindering cell death mechanisms, such as apoptosis and necroptosis, particularly within the extrinsic pathway [289]. cFLIP is present in the three splice variants named cFLIPL (FLIP long), cFLIPS (FLIP short), and cFLIPR (FLIP Raji) [290]. cFLIPL bears a resemblance to caspase-8 in structure, but lacks enzymatic activity due to several alterations in crucial catalytic amino acids, functioning as a primary inhibitor by obstructing the activation of pro-caspase 8 and preventing the release of active caspase-8 from the DISC [289, 291]. Necroptosis occurs when the activation of caspase-8 is blocked, leading to a type of programmed necrotic cell death triggered by the interplay of RIP-1 and RIP-3 [292]. The control of necroptosis relies on the particular type of cFLIP, with cFLIPL blocking ripoptosome formation and cFLIPS aiding its assembly, ultimately resulting in cell death [293]. cFLIPL inhibits autophagy by directly interacting with the autophagy machinery, specifically by competing with Atg3 for binding to LC3. This competition results in reduced LC3 processing, leading to the inhibition of autophagosome formation [293]. It is prominently present in the cardiac tissue, thus playing an important role in maintaining cellular homeostasis within cardiac tissue. During ischemia/reperfusion injury, the downregulation of cFLIPL expression results in increased endoplasmic reticulum stress and apoptosis within cardiac tissue [294, 295]. The expression of cFLIP also decreased in the cardiomyopathic heart and promoted cell death of cardiac tissue [296]. The myocardium of a rat experiencing cardiotoxicity showed a reduction in cFLIPL expression when exposed to adriamycin, leading to cell death in heart tissue. Moreover, adriamycin increased NF-κβ expression, inducing inflammation in cardiac tissue, a response mitigated by co-administrating fusodil [290]. Dox triggered Fas-mediated apoptosis in cardiomyocytes by inducing dose- and time-dependent downregulation of cFLIPL expression through the generation of ROS in cardiac tissue [297]. However, administering Dox at a dose of 10 mg/kg did not change the expression of cFLIP protein in cardiac tissue, suggesting that this pathway may not be involved in the apoptotic cardiac muscle or may not be affected at this dose [298]. In an *in vitro* study, Dox did not change cFLIP expression in H9c2 cells, showing the Dox-mediated apoptotic process to be independent of Fas/FasL signaling [299]. cFLIPL and cFLIPS have diverse functions in different signaling pathways and these can activate or increase the activity of several pathways that promote cell protection and survival, such as Ak, MAPK, JNK, NF-κβ, and Wnt [293, 300, 301]. The mechanism of cFLIP in anthracycline-induced cardiotoxicity is not well-known, so it is essential to conduct more study to conclude the precise mechanism of cFLIP in the pathophysiology of anthracycline-induced cardiotoxicity. Additionally, an exploration of its crosstalk with other signaling molecules is also required through preclinical studies in the future so that it may prove to be a potential target for the management of cardiotoxicity.

### 3.14 XIAP (X-linked Inhibitor of Apoptosis)

XIAP is a protein that inhibits apoptosis; it belongs to a group of proteins that have a consistent evolutionary function in controlling programmed cell death and acts as a downstream mediator of the PI3K/Akt pathway. It reduces the action of caspase-9, a key inhibitor in the extrinsic pathway, and also targets caspase-3, which is a common mediator in both the extrinsic and intrinsic pathways [302]. The mitigation of apoptosis induced in cardiomyocytes following cardiac damage after HF could be achieved by the induction of XIAP. This upregulation effectively suppressed the activity of caspase-3/7, suggesting a promising therapeutic approach for HF treatment [303]. Furthermore, XIAP-mediated downregulation of cleaved caspase3 and Bax and overexpression of Bcl-2 could directly arrest the development of acute myocardial infarction [304]. The application of Dox has been demonstrated to decrease cFLIP and XIAP levels by increasing NF-κβ expression, while also boosting the presence of TNF-related Apoptosis-inducing Ligand receptors (TRAIL), thus initiating and progressing apoptosis [103]. Furthermore, the Ultraviolet radiation Resistance-associated Gene (UVRAG), a protein involved in autophagy, is vital for sustaining the continuous process of autophagy in the heart under normal conditions [305]. The lack of UVRAG intensified both mortality and heart damage induced by Dox, as evidenced by an increase in cytoplasmic vacuolization, more collagen buildup, higher levels of LDH and creatine kinase in the bloodstream, elevated levels of ROS, increased apoptosis, and retarded cardiac function [306]. Mechanistically, Dox-treated UVRAG-deficient mice showed a decrease in the XIAP levels, elevated levels of TNF-α, IL-6, and IL-1β, and increased LC-II and Beclin protein levels, resulting in cardiac dysfunction [306]. This pathway might be a novel target in the DIC by inhibiting apoptosis, needing further exploration in the design of an intervention strategy (Fig. **4**).

### 3.15 p53 Pathway

The p53 pathway functions as a key mechanism for suppressing tumors by mediating essential cellular processes, including the repair of DNA, regulation of the cell death and cell cycle, and induction of cellular senescence [307]. Inhibition of p53 induces autophagy across human, mouse, and nematode cells, while diverse autophagy stimuli contribute to p53 breakdown. Conversely, preventing the degradation of p53 hinders autophagy, indicating that inhibiting p53 is crucial for inducing autophagy [308]. P53 exerts a dual influence on autophagy, with nuclear p53 stimulating autophagy through a transcriptional mechanism while cytoplasmic p53 serving as a primary suppressor of autophagy [309]. In normal conditions, the basic amount of p53 (p53b) hinders AMPK to ease the inhibition of autophagy caused by mTORC1. This impact of mTORC1 involves its interaction with FIP200 in influencing the formation of the ULK1/2 complex. When stress triggers the activation of p53, it relocates to the nucleus to promote the expression of autophagy genes, including AMPK, DRAM1, PTEN, and sestrins, yet the resultant autophagy does not confer cardioprotective effects [310]. In conditions of elevated stress, if the strength of signals promoting cell death surpasses the cell’s defense mechanism, the movement of p53 to the mitochondria initiates the permeabilization of the mitochondrial outer membrane, leading to the upregulation of the mitochondrial apoptotic pathway [311]. The levels of p53 rise in the heart during stress-induced injuries through both transcription-independent and transcription-dependent pathways. The transcription-dependent pathways control mitochondrial signals for cell death, increasing genes that promote apoptosis, like NOXA, Bax, and PUMA, and reducing genes that inhibit apoptosis, like Bcl-2 [312]. A research study mentioned that p53 plays a role in the increased cardiac toxicity caused by Dox. In a study, P53 wild-type and knockout C57BL mice were administered Dox; ejection fraction was observed to be reduced in the wild-type and cell death was observed to be increased *via* apoptosis, while no changes were demonstrated in the knockout mice [313]. Furthermore, p53 protein was reported as accountable for hindering the progression of the cell cycle and activating intrinsic and extrinsic pathways, which are considerable mechanisms contributing to the initial stages of DIC [314]. An *in vitro* study elucidated that the impaired functioning of mitochondria in H9c2 myoblasts exposed to Dox emerged from the activation of nuclear p53, rather than being a direct outcome of the drug’s impact on mitochondria. The study findings demonstrated that Dox treatment of cardiac cells increased p53 protein and Bax protein expressions in the mitochondria, further inducing apoptosis and cell death [315]. In a pre-clinical study, it was estimated that Dox triggered the JNK-p38 and p53 signaling pathways, leading to heart damage in rats through oxidative stress and cell death. All these effects of Dox were reversed by the administration of arjunolic acid in the rats, which increased cardiomyocyte viability [316]. Retinoic acid, a natural derivative of vitamin A, showed cardioprotective effects by reducing oxidative stress, inflammation, and apoptosis, by suppressing the p53 signaling pathway [317]. Table **6** includes some further studies on this pathway.

### 3.16 JAK-STAT Signaling Pathway

Janus Kinase/Signal Transducer and Activator of Transcription (JAK-STAT) signaling is essential for immunological responses, differentiation, and cell proliferation. The pathway starts with Janus Kinases (JAKs), receptor-associated tyrosine kinases with four key members, named JAK1, JAK2, JAK3, and TYK2 [318, 319]. When the ligand attaches to its receptor, it triggers the pairing of receptors, activating JAKs. These JAKs then add a phosphate group to tyrosine residues on both the receptor and themselves, creating sites where downstream signaling molecules can bind [319]. The receptor complex recruits inactive transcription factors STAT1, STAT2, STAT3, STAT4, STAT5a, STAT5b and STAT6. Upon being recruited and phosphorylated by JAKs, STATs assemble into homodimers or heterodimers, and then migrate to the cell nucleus where they act as transcription factors. STAT dimer attaches to its target gene promoter DNA sequences in the nucleus, inducing the transcription of genes that encode proteins associated with immunological modulation, differentiation, and cell proliferation [320]. Numerous genes stimulated through the JAK-STAT pathway produce inflammatory proteins, such as chemokines (IL-8), cytokines (IL-6, TNF-α), and acute-phase proteins, which attract immune cells to the inflammation site [321, 322]. These inflammatory proteins produced as a result of JAK-STAT activation attract immune cells to the site of inflammation. The recruited immune cells further contribute to inflammation by releasing more cytokines and chemokines, perpetuating the inflammatory response [323, 324]. However, elevated levels of STAT3 provided protective effects against DIC in mice. Conversely, impeding STAT3 function through the JAK2 inhibitor AG-490 led to a rise in TUNEL-positive cells (indication of apoptosis) after I/R injury [325]. However, the JAK2 and STAT3 expression was suppressed after treatment of Dox in the cardiomyocytes model. Knockdown of these proteins inhibited autophagy and increased mitochondrial ROS, which increased cell death in cardiomyocytes [326]. It has been found that Dox decreased the expression of STAT3 and increased STAT1 expression, but it did not change the activity of JAK1 and JAK2. The results of these alterations reduced ERK1/2 activity and enhanced p38 activity, leading to apoptosis in cardiomyocytes. Suppression of the JAK/STAT3 pathway with Dox hindered the transcription of genes that prevent cell death, ultimately leading to a negative impact on cardiac outcomes [327]. In contrast, a recent study found that Dox administration partially activated the JAK2/STAT3 pathway in the Sprague-Dawley rats. This partial activation had been enhanced by treatment with fucoidan and protected against DIC by increasing autophagy and preventing apoptosis in the JAK2/STAT3-dependent manner [328]. The induction of cardioprotection has been demonstrated through activation of JAK/STAT regulated by IL-6, as the expression of STAT3 is intrinsically upregulated in the myocardium. However, an excessive increase in its expression might result in the development of cardiomyopathy [103]. The upregulation of the JAK/STAT pathway has been linked with various diseases, such as HF, fibrosis, hypertension, and myocarditis, and inhibition of this pathway has been found to enhance the restoration of heart function [329, 330].

### 3.17 Nucleotide-binding Domain-like Receptor Protein 3 (NLRP3) Inflammasome Activation

The NLRP3 inflammasome comprises a complex arrangement of regulatory proteins that collaborate to trigger an inflammatory reaction. When activated, the NLRP3 inflammasome initiates a cascade of cellular processes that cause the recruitment of apoptosis-associated speck-like protein containing a carboxyterminal caspase recruitment domain and caspase-1, ultimately resulting in the generation and release of inflammatory signaling molecules, like IL-1β and IL-18 [331, 332]. Upon cardiac injury, the upregulation of the NLRP3 inflammasome in the heart results in immediate local consequences. Specifically, IL-1β and IL-18 mechanistically transmit signals through the transmembrane IL-1 type I Receptor (IL-1R1), thereby amplifying the damage to the heart. For instance, when IL-1 activates IL-1R1, it initiates the death of heart muscle cells, leads to more loss of muscle fibers, decreases the sensitivity of β-adrenergic receptors, diminishes the heart's ability to contract, and ultimately causes adverse effects on the cardiac physiology [333]. Increasing evidence indicates that the activation of the NLRP3 inflammasome and subsequent release of proinflammatory cytokines play a crucial part in the onset of cardiotoxicity induced by Dox [334-336]. The administration of Dox led to an increase in NLRP3 expression, resulting in the production of inflammatory cytokines IL-1β and IL-10 in cardiac muscles. This suggests that the NLRP3/IL-1β pathway may play a role in the development of DIC [360]. A study found that the lack of NLRP3 heightened cardiac dysfunction and injury induced by Dox independently of IL-1β in NLRP3−/− and IL-1β−/−mice [337]. Another research investigation verified that MCC950, a substance that inhibits the NLRP3 inflammasome, could reduce heart damage caused by Dox both in lab experiments and in living organisms by blocking a process called NLRP3-mediated pyroptosis [338]. According to reports, the absence of NLRP3 increased vulnerability to heart toxicity regardless of IL-1β. This was shown in a study where Dox-induced cardiac dysfunction and damage occurred in NLRP3-/-mice, but not in wild-type or IL-1β-/-mice [337]. The restriction of NLRP3 hindered apoptosis in primary heart muscle cells caused by Dox, highlighting the significant involvement of ROS/NLRP3 inflammasome activation in DIC. Moreover, Dox caused the programmed cell death of H9c2 cells and primary cardiomyocytes by increasing the expressions of NLRP3, ASC, and caspase-1 p20, along with elevated secretion of IL-1β, indicating the activation of the NLRP3 inflammasome [339]. A study assessed SIRT3's role in DIC in C57BL/6 mice. Boosting SIRT3 levels significantly reduced DIC by suppressing the NLRP3 inflammasome. This suggests that enhancing or activating SIRT3 could be a promising strategy for treating DIC [340]. Nevertheless, activation of SIRT1 has been documented to adversely control inflammation dependent on the NLRP3 inflammasome [341]. In an investigation, the stimulation of dopamine D1 receptors by A-68930, a particular activator, reduced the expression of NLRP3 and activation of caspase-1 and maturation of IL-1β caused by Dox in H9c2 cells. The findings suggest that the signaling of dopamine D1 receptors might shield against Dox-triggered heart damage by suppressing inflammation mediated by the NLRP3 inflammasome (Table **6**) [342].

### 3.18 Ferroptosis

Ferroptosis is a recently discovered mechanism of cell death that occurs due to the accumulation of iron and the oxidation of lipids within cells. This process entails a reduction in the levels of antioxidant enzymes, leading to the occurrence of lipid peroxidation and oxidative stress. It is a form of cell death that is triggered by iron and oxidative damage. This process involves specific changes in the cell's structure, including a decrease in the number of folds in the mitochondria and damage to the membranes of the mitochondria. The cell abnormalities are caused by the disruption of the plasma membrane's ability to selectively allow certain substances to pass through as a result of the extensive oxidation of membrane lipids and the presence of oxidative stress. There have been numerous studies [189, 354, 355] that have indicated the role of ferroptosis-mediated cardiotoxicity by anthracyclines. DOX can promote ferroptosis in cardiomyocytes by modulating iron homeostasis-related proteins and Iron-responsive Elements (IREs)/Iron Regulatory Proteins (IRPs), resulting in elevated iron levels in cardiomyocytes. Additionally, DOX can increase ROS, causing cell membrane lipid peroxidation. The Nrf2 signalling pathway plays a vital function. As the major organelle for ROS generation and the site where iron accumulation may occur, mitochondria are critical for developing DOX-induced cardiomyopathy [356]. For instance, in DOX-induced cardiotoxicity in mice, treatment with fucoidan (fucose-containing sulfated polysaccharides) improved cardiac functions by decreasing serum creatine kinase and lactate dehydrogenase, as well as reduced levels of lipid ROS, GSH, MDA, and ferroptosis-related markers and regulatory factors, like GPX4, transferrin receptor protein-1, ferritin heavy chain-1, and HO-1 in the heart tissue. Additionally, *in vitro* studies have revealed that this reduced ferroptosis can be due to the activation of Nrf2, which increases the activity of GPX4 [357]. Moreover, some other mechanisms have been reported through which anthracyclines can induce cardiotoxicity *via* ferroptosis. For example, DOX treatment in AMPKα2 knockout mice caused cardiac dysfunction, increased mortality, and mitochondrial injury, and increased the expression of ferroptosis-associated proteins and genes, resulting in lactate dehydrogenase and MDA accumulation in hearts. The study has found AMPKα2 knockout mice to have higher levels of Polyunsaturated Fatty Acids (PFAs), oxidised lipids, and Phosphatidylethanolamine (PE), associated with ferroptosis. Therefore, DOX induced cardiotoxicity by altering the AMPK/ferroptosis pathway [358]. Similarly, epirubicin also has been reported to induce cardiotoxicity *via* disruption of ATP6V0A2-dependent lysosomal acidification, thus triggering ferroptosis in cardiomyocytes. In this study, results obtained from qRT-PCR revealed the downregulation of ATP6V0A2 (which is essential to maintain lysosomal acidification to suppress ferroptosis) in neonatal primary mouse ventricular cardiomyocytes, subjected to epirubicin [359]. Mitochondria are the primary organelles responsible for inducing ferroptosis in cardiomyocytes, and it has been discovered that DOX-induced cardiotoxicity *via* ferroptosis is due to mitochondrial malfunction. For example, DOX-induced cardiotoxicity in C57BL/6 mice caused an increase in myocardial damage markers, such as cTNT, CK-MB, ANP, and BNP, as well as an increase in ferroptosis-associated markers, such as Fe2+ and MDA, and a reduction in GPX4 levels (a ferroptosis regulator). Furthermore, transmission electron microscopy demonstrated the DOX-treated group to have vacuolation, disorganised and lose arrangement of cristae, and an incomplete membrane in the mouse mitochondria. Furthermore, ATP generation and mitochondrial membrane protein TOM20 expression levels in myocardial samples were all significantly lowered following DOX treatment, as well as there were decreased levels of GSH, indicating an increase in oxidative stress [360]. These investigations show the crucial involvement of ferroptosis in anthracycline-induced cardiotoxicity and prospective treatment options that target this pathway to reduce cardiac damage.

Furthermore, several ferroptosis inhibitors have demonstrated cardioprotective effects against anthracycline-induced cardiotoxicity, such as ferrostatin-1, a synthetic anti-oxidant that suppresses ferroptosis by scavenging alkoxyl radicals and decreasing the ferrous ion radical. Ferrostatin-1 therapy significantly decreased cardiomyopathy and oxidative stress, and inhibited ferroptosis in DOX and ischemia/reperfusion-induced cardiotoxicity canonical apoptosis and/or necroptosis-defective Ripk^3−/−^, Mlkl^−/−^, or Fadd^−/−^Mlkl^−/−^ mice [361]. The administration of melatonin, a hormone and antioxidant, and deferoxamine, an iron chelator that binds iron and aluminium, also inhibited ferroptosis in DOX-induced acute cardiotoxicity. This was demonstrated by elevated GPX4 expression, ferritin heavy chain (FTH1), antioxidant elements (GSH), and decreased MDA levels [362]. Furthermore, treatment of N-acetylcysteine was reported to improve cardiac functions by reducing ferroptosis, as evidenced by increased expression of GPX4 and ameliorated DOX-induced cardiotoxicity in MITOL knockout rats [363]. It has been observed that various additional ferroptosis inhibitors can lessen the cardiotoxicity caused by anthracycline, as compiled in Table **7** [364-368].

## CONCLUSION

The primary clinical treatment approaches for Doxorubicin-induced Cardiotoxicity (DIC) involve minimizing the total dose of Dox administered, utilization of liposomal formulation of Dox, and employing cardioprotective drugs, including dexrazoxane, ACE inhibitor, β-blockers, angiotensin receptor blocker, statins, and mineralocorticoid receptor antagonist [15, 369]. In recent findings, 82% of patients displaying indications of cardiotoxicity during first-year post-anthracycline treatment demonstrated a substantial or partial improvement in LVEF. This positive outcome was observed in individuals shortly treated with the ACE inhibitor enalapril, either as a single drug or in conjunction with carvedilol [370]. Nevertheless, it remains uncertain whether the improvements in heart function or direct protection effects contributed to these results, along with the preservation of the effectiveness of cancer treatment. Consequently, the suitable application of such drugs in the cancer framework remains controversial, and identifying the optimal cardioprotective approach for individuals with cancer remains an unaddressed clinical requirement [371]. Dexrazoxane stands as the only approved medication for preventing DIC, acting by inhibiting topoisomerase II and the Dox-Fe^2+^ complex [372]. Furthermore, dexrazoxane has the capacity to mitigate Dox-induced cell death through the enhancement of miR-17-5p expression and the inhibition of the p38 Mitogen-activated Protein Kinase (p38 MAPK)/Nuclear Factor-kappa B (NF-κβ) pathway [373, 374]. A study indicated that over 59% of patients who underwent dexrazoxane pretreatment exhibited increased levels of troponin T, which implies that dexrazoxane pretreatment may not entirely resolve DIC [375].

Anthracycline-based chemotherapy remains a crucial component in the treatment of various cancers. However, its clinical application is often hampered by the significant risk of developing cardiotoxicity, resulting in detrimental consequences for patients. The specific processes that underlie anthracycline-induced cardiotoxicity are multifaceted, including a complicated interaction between many signaling pathways. This review has comprehensively explored the current understanding of these mechanisms, encompassing oxidative stress, inflammatory response, mitochondrial dysfunction, autophagy, apoptosis, Ca^2+^ overload, and endoplasmic reticulum stress through PI3K/Akt, mTOR, ULK1, FOXO, GSK-3, MAPK/ERK, Nrf2/Keap1, NOTCH, Wnt/β-catenin, TLR, NF-κβ, IRF, cFLIP, XIAP, p53, JAK-STAT, and NLRP3 signaling pathways. By elucidating these signaling pathways, we gain valuable insights into the development and progression of anthracycline-induced cardiotoxicity. Despite the substantial progress made in comprehending the mechanisms responsible for anthracycline-induced cardiotoxicity, there remains a compelling need for further investigation in several critical areas. Additionally, the precise contributions of different signaling pathways as well as their possible interconnections require more research. However, identifying novel therapeutic targets within these pathways holds immense promise for the development of more efficacious preventative and treatment strategies. The specific areas for future research can include investigating the potential of targeting specific molecules within the identified signaling pathways to prevent or mitigate cardiotoxicity and exploring the potential benefits of combining conventional cardioprotective agents with targeted therapies directed at specific signaling pathways. Furthermore, the findings obtained from preclinical studies can be translated into clinical trials in order to evaluate the efficacy and safety of novel therapeutic approaches in managing anthracycline-induced cardiotoxicity.

## Figures and Tables

**Table 1:** Anthracyclines - structures, mechanisms, indications, and toxicity profiles.

**S. No.**	**Drug**	**Structure**	**Mechanism of Action**	**Indications**	**Toxicity Profile**
1.	Doxorubicin	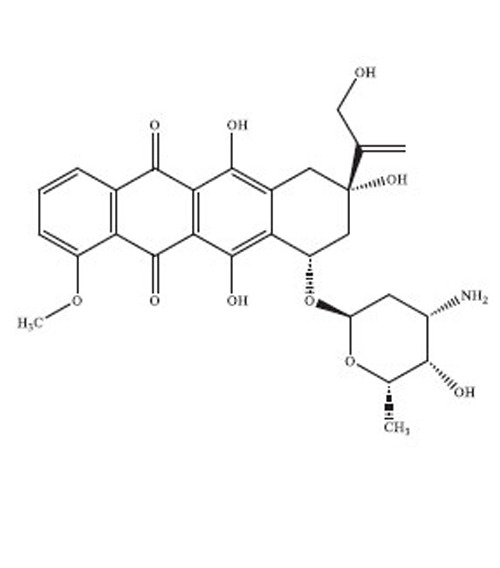	• *DNA intercalation*: intercalates between base pairs in the DNA helix → disruptions of DNA structure → disruption of replication and transcription • *Inhibition of topoisomerase II*: stabilizes the DNA-topoisomerase II complex → preventing re-ligation of DNA strands • *ROS generation*: oxidative damage to cellular components → cytotoxic effects on cancerous cells. • *Cell membrane permeability*: interacts with membrane lipids → disruption of membrane permeability → altered functions → cell death. • *Apoptosis induction*: activation of apoptotic pathways → due to cellular damage → programmed cell death [28].	• Breast cancer • Acute Lymphoblastic Leukemia (ALL) • Hodgkin’s lymphoma • Non- Hodgkin’s lymphoma • Ovarian cancer • Bladder cancer • Acute Myeloid Leukemia (AML) • Thyroid cancer • Gastric cancer • Smal cell lung cancer [28, 29].	• Cardiotoxicity • Myelosuppression • Gastrointestinal toxicity • Alopecia • Local tissue damage • Hepatotoxicity • Immunosuppression [28, 30]
2.	Daunorubicin	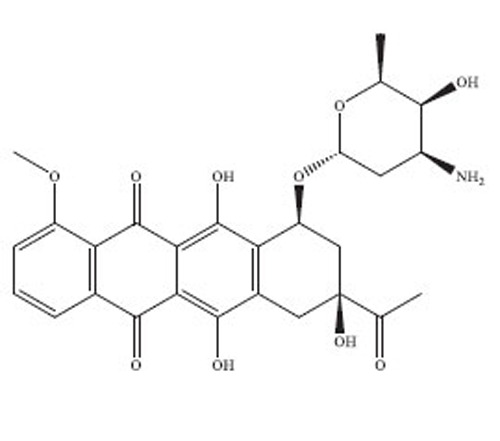	• *DNA intercalation*: intercalates between base pairs in the DNA helix → disruption of DNA structure → disruption of replication and transcription. • *Inhibition of topoisomerase II*: stabilizes the DNA-topoisomerase II complex → preventing re-ligation of DNA strands. • *ROS generation*: oxidative damage to cellular components → cytotoxic effects on cancerous cells. • *Cell membrane permeability*: interacts with membrane lipids → disruption of membrane permeability → altered functions → cell death. • *Apoptosis induction*: activation of apoptotic pathways → due to cellular damage → programmed cell death [28].	• Acute Lymphoblastic Leukemia (ALL) • Acute Myeloid Leukemia (AML) • Chronic myelogenous leukemia • Myelodysplastic syndrome • Neuroblastoma • AIDS-related Kaposi's sarcoma [31-34]	• Cardiotoxicity • Myelosuppression • Gastrointestinal toxicity • Alopecia • Local tissue damage • Hepatotoxicity • Immunosuppression [31]
3.	Epirubicin (4'-epi-isomer of doxorubicin)	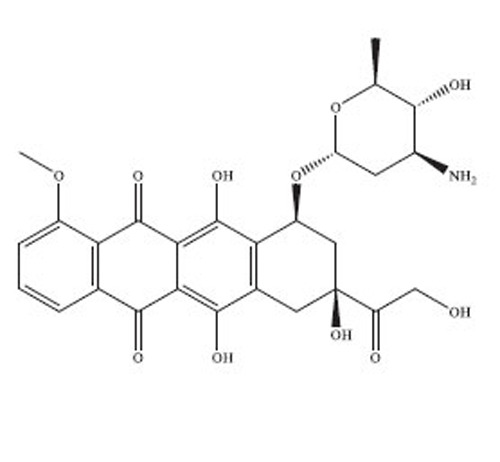	Same as doxorubicin and daunorubicin; however, it is less potent than doxorubicin, but has a somewhat low cardiotoxicity profile [35].	• Gastric cancer • Breast cancer • Ovarian cancer • Non-Hodgkin’s cancer • Bladder cancer • Esophageal cancer • Small cell lung cancer • Soft tissue sarcomas • Pancreatic cancer • Head and neck cancer [25, 35-37]	• Myelosuppression • Gastrointestinal toxicity • Cardiotoxicity • Alopecia • Extravasation and local tissue damage • Fatigue • Hepatotoxicity • Immunosuppression • Hand-foot syndrome • Changes in urine colour [38, 39]
4.	Idarubicin (4′-demethoxydaunorubicin)	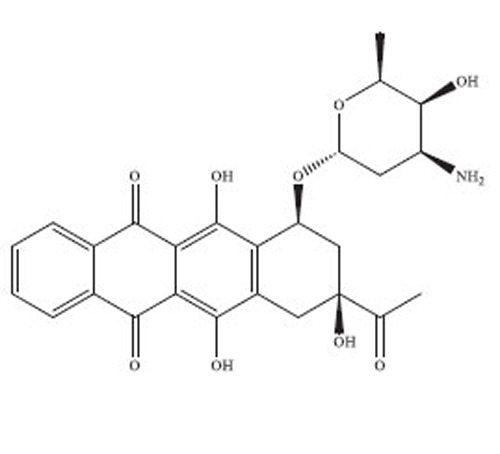	Same as doxorubicin and daunorubicin; however, it is considered more potent than doxorubicin [40].	• Acute Lymphoblastic Leukemia (ALL) • Myelodysplastic Syndrome (MDS) • Acute Promyelocytic Leukemia (APL) • Relapsed or refractory leukemias • Acute Myeloid Leukemia (AML) • Chronic myelogenous leukemia [41, 42]	Similar toxicity profile as doxorubicin; however, idarubicin has a slightly lower risk of cardiotoxicity in comparison to doxorubicin, and has more profound and prolonged myelosuppression compared to doxorubicin. It has a longer half-life and different tissue distribution in comparison to doxorubicin [41, 43]
5.	Mitoxantrone (dihydroxyanthraquinone/anthracenedione antineoplastic agent)	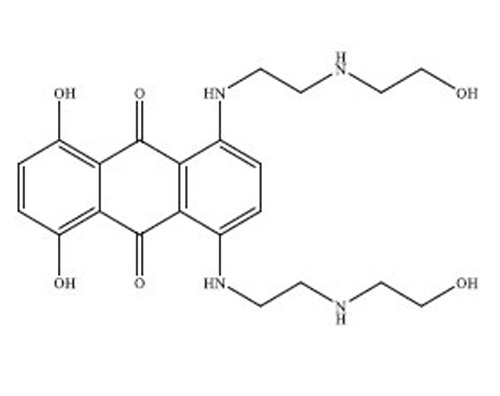	Similar mechanism of action as doxorubicin, but has an additional RNA disruption mechanism in which it disrupts the RNA synthesis and function → impaired protein synthesis → cell growth inhibition [44, 45].	• Acute Myeloid Leukemia (AML) • Advanced breast cancer • Hormone-refractory prostate cancer • Non-Hodgkin's lymphoma • Multiple sclerosis [46, 47]	It has a similar toxicity profile as doxorubicin; however, it leads to the following side effects: • Renal toxicity • Blue-green discolouration of urine, sclera, and skin • Menstrual irregularities and infertility • Allergic reactions Also, it has less distribution in tissues as compared to doxorubicin [48, 49]
6.	Valrubicin (N-trifluoroacetyl, 14-valerate derivative of the anthracycline doxorubicin)	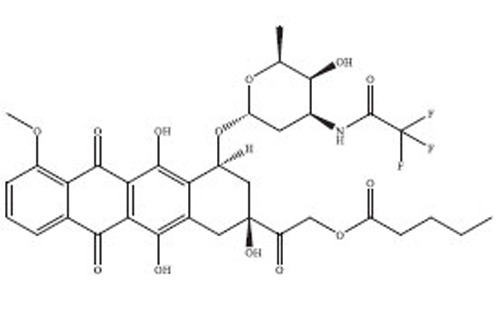	Similar mechanism of action as doxorubicin [50].	• Non-muscle invasive bladder cancer [50, 51]	• Local bladder toxicity: bladder irritation, hematuria, cystitis • Urinary tract infection • Urinary retention • Bladder spasms • Allergic reactions [52]
7.	Aclarubicin	-	Similar mechanism of action as doxorubicin [53].	• AML • ALL • Hematological cancers [53]	• Myelosuppression • Gastrointestinal toxicity • Hepatotoxicity • Renal toxicity • Allergic reactions • Lower cardiotoxicity as compared to other anthracyclines [53]
8.	Pirarubicin	-	Similar mechanism of action as doxorubicin [54].	• Breast cancer • Bladder cancer [55]	• Myelosuppression • Gastrointestinal toxicity • Hepatotoxicity • Renal toxicity • Allergic reactions [56]
9.	Zorubicin	-	Similar mechanism of action as doxorubicin [57].	• Breast cancer • Hematologic malignancies [58]	Same as other anthracyclines but with efforts to reduce side effects while maintaining antitumor efficacy [57]
10.	Amrubicin		Similar mechanism of action as doxorubicin [59].	• Small cell lung cancer • Non-small cell lung cancer [60]	Same as other anthracyclines, but having less cardiotoxicity [61]

**Table 2:** Various drugs showing protective action against anthracycline-induced cardiotoxicity by regulation of the PI3K/Akt/TFEB/mTOR/GSK-3β and AMPK/mTOR pathways.

**S. No.**	**Therapeutic Agent**	**Preclinical Model**	**Molecular Mechanism**	**Outcome**	**References**
1.	Paeonol	Epirubicin-induced cardiotoxicity in H9c2 myocytes	Suppression of PI3K/Akt/mTOR and NF-ĸβ pathways	↓ Inflammation and apoptosis ↑ Autophagy	[145]
2.	Benazepril hydrochloride	DIC in H9c2 cell line	Activation of PI3K/Akt	↑ CAT, GSH, and SOD ↓ Apoptosis	[146]
3.	Propofol	Adriamycin-induced cardiotoxicity in rats	Suppression of PI3K/Akt/Bcl-2 pathway	↓ Troponin and BNP levels ↓ Apoptosis	[147]
4.	Micheliolide	DIC in mice	Inhibition of PI3K/Akt/NF-ĸβ pathways	↓ TNF-α, NF-κβ, and IL-1β expressions ↑ SOD and GPx levels ↓ Troponin and CK-MB levels	[148]
5.	Astragalus polysaccharide	DIC in C57BL/6J mouse	Activation of PI3K/Akt pathway	↑ Cell survival ↓ Oxidative stress and inflammation	[149]
6.	Apigenin	Adriamycin-induced cardiotoxicity	Activation of PI3K/Akt/mTOR pathway	↓ AST, LDH, and CK-MB levels ↓ Beclin1 and LC3B II/I expression ↓ Bax/Bcl-2 ratio	[150]
7.	Qishen Huanwu capsule	Pirarubicin-induced cardiotoxicity in rats	Activation of the PI3K/Akt/mTOR pathway	↑ LVEF ↑ p-mTOR, p-Akt, and p-PI3K expression	[151]
8.	Wheat phenolics	DIC in rats	Activation of PI3K/Akt/mTOR pathway Inhibition of p38/JNK/MAPK signaling pathway	↑ AREs ↓ Levels of Fas, Bid, NF-κβ, and caspase 8 ↓ LDH, CK-MB, and AST levels	[152]
9.	Higenamine combined with 6-gingerol	DIC in H9c2 cell line	Activation of PI3K/Akt pathway	↓ ROS generation and apoptosis ↑ p-Akt ↑ Cell survival	[153]
10.	Dapagliflozin	DIC in H9c2 cell line	Modulation of the PI3K/Akt/Nrf2 pathway	↑ Antioxidant capacity ↓ Activation of NF-κβ p65 ↓ Inflammation	[154]
11.	Eupatilin	DIC in mice	Activation of PI3K/Akt pathway	↓ Oxidative stress and inflammation ↓ Apoptosis	[155]
12.	Xinmailong	Epirubicin-induced cardiotoxicity in rats	Activation of PI3K/Akt pathway	↓ Beclin1 and Atg7 expression ↓ P38 and Erk1/2	[156]
13.	Kirenol	DIC in the H9c2 cell line	Activation of PI3K/Akt and Nrf2 pathways	↓ MMP-2 and MMP-9 levels ↑ p53 expression ↓ MDA levels	[157]
14.	Liraglutide	DIC in rats	Modulation of Akt/GSk-3β pathway	↓ Troponin and CK-MB levels ↓ MDA, IL-6, TNF-α, and caspase-3 levels ↑ SOD activity	[158]
15.	Asiatic acid	DIC in mice	Activation of the Akt/GSk-3β pathway	↓ Troponin, CK-MB, and LDH levels ↑ SOD, GSH, and CAT levels	[159]
16.	Dexmedetomidine	DIC in H9c2 cells	Upregulation of the AMPK/GSK-3β pathway	↓ TNF-α, IL-1, and IL-6 levels ↓ TXNIP, NLRP3, and ASC expressions	[160]

**Table 3:** Pharmacological agents demonstrating cardioprotective efficacy *via* activation of Nrf2/Keap1/ARE and inhibition of MAPK/ERK signaling pathways in anthracycline-induced cardiotoxicity preclinical studies.

**S. No.**	**Therapeutic Agent**	**Preclinical Model**	**Molecular Mechanism**	**Outcome**	**References**
1.	Cardamonin	DIC in mice	Activation of Nrf2 pathway	↑ NAD(P)H, SOD, GSH and CAT expressions ↓ Oxidative stress	[193]
2.	Ginsenoside Rg3	Adriamycin-induced cardiotoxicity in rats	Activation of the Nrf2/ARE pathway	↓ Apoptosis ↑ AREs	[194]
3.	Cafestol	DIC in rats	Activation of the Nrf2 pathway	↓ CK-MB and LDH levels ↑ CAT, GSH, and SOD levels ↓ TNF-α and IL-1β levels	[195]
4.	Tanshinone IIA	DIC in mice	Activation of the Nrf2 pathway	↓ AST, LDH, and CK-MB levels ↓ Oxidative stress	[196]
5.	α-linolenic acid	DIC in rats	Activation of the Nrf2 pathway	↑ LVEF ↓ Apoptosis and oxidative stress ↑ Nrf2 and SODmRNA	[197]
6.	Asiatic acid	DIC in rats	Activation of the Nrf2 pathway	↓ CK-MB and LDH levels ↑ AREs ↓ Apoptosis	[198]
7.	Orosomucoid 1	DIC in mice	Stimulation of Nrf2 signaling	↓ Oxidative stress ↓ Apoptosis	[199]
8.	Baicalein	DIC in mice	Stimulation of the Nrf2 pathway	↓ CK-MB and LDH levels ↓ Oxidative stress ↓ Apoptosis	[200]
9.	Metformin	DIC in Sprague-Dawley rats	Stimulation of the AMPK pathway Suppression of the MAPK pathway	↓ Cardiac injury ↓ Apoptosis ↓ Oxidative stress	[201]
10.	Chitosan oligosaccharides	DIC in Sprague-Dawley rats	Suppression of the MAPK pathway	↓ Oxidative stress ↓ MMP and Bcl/Bax ratio	[202]
11.	Fucoxanthin	DIC in mice	Suppression of the MAPK pathway	↓ Oxidative stress ↓ Apoptosis	[203]
12.	Hydrogen sulfide	DIC in H9c2 cells	Suppression of the MAPK pathway	↓ Apoptosis ↓ Oxidative stress ↑ MMP	[204]
13.	Ghrelin	DIC in mice	Suppression of the MAPK pathway	↑ AREs ↓ Autophagy	[205]
14.	Myricitrin	DIC in rats	Inhibition of the ERK pathway	↑ MMP ↓ Caspase-3 and Bcl/Bax ratio	[206]
15.	Chrysin	DIC in rats	Inhibition of the ERK pathway	↓ CK-MB and LDH levels ↑ AREs ↓ Caspase-3	[207]
16.	Luteolin-7-O-glucoside	DIC in H9c2 cells	Suppression of PTEN/Akt and ERK pathways	↓ Caspase-3 and Bcl/Bax ratio ↑ AREs	[208]
17.	Rosmarinic acid	Adriamycin-induced cardiotoxicity in H9c2 cells	Suppression of the ERK pathway	↓ Oxidative stress ↓ Apoptosis ↑ MMP	[209]
18.	Hydrogen sulfide	DIC in H9c2 cells	Suppression of the ERK pathway	↓ Oxidative stress ↓ Apoptosis	[210]
19.	Kaempferol	DIC in rats	Inhibition of the ERK1/2 pathway	↓ Oxidative stress ↓ Apoptosis	[211]
20.	Urotensin II	DIC in rats	Inhibition of the ERK1/2 pathway	↓ Oxidative stress ↓ Apoptosis	[212]
21.	Davallialactone	Adriamycin-induced cardiotoxicity in H9c2 cells	Inhibition of ERK and JNK pathways	↓ Oxidative stress ↓ Apoptosis	[213]

**Table 4:** Pharmacological agents exhibiting cardioprotective effects against anthracycline-induced cardiotoxicity through upregulation of the NOTCH pathway and Wnt/β-catenin signaling.

**S. No.**	**Therapeutic Agent**	**Preclinical Model**	**Molecular Mechanism**	**Outcome**	**References**
1.	Beetroot juice	DIC in mice	Upregulation of NOTCH1 mRNA/protein expression	↑ Expression of the cardiac microRNA 34a, Sirt1, and NOTCH1	[241]
2.	Yangxin	DIC in H9c2 cardiomyocytes	Activation of β-catenin signaling	↓ Apoptosis ↓ Inflammation and oxidative stress	[236]
3.	Baicalin	DIC in mice	Activation of β-catenin signaling by suppressing Dkk-1	↓ Oxidative stress ↓ Inflammation and apoptosis	[242]

**Table 5:** Pharmacological agents mitigating anthracycline-induced cardiotoxicity through TLR receptor suppression.

**S. No.**	**Therapeutic Agent**	**Preclinical Model**	**Molecular Mechanism**	**Outcome**	**References**
1.	Metformin	DIC in mice	Suppression of HMGB1/TLR4/NLRP3 pathway	↓ Oxidative stress ↓ BNP, CK-MB, and troponin-I levels ↓ Inflammation and apoptosis	[265]
2.	Vanillic acid	DIC in rats	Suppression of the TLR4 pathway	↓ LDH, MDA, CK-MB, and troponin-I levels	[266]
3.	Sheng-Mai Yin	DIC in rats	Inhibition of the TLR2 pathway	↓ BNP and Ck-MB ↓ TGF-β level ↓ MCP-1, IL-6, INF-γ	[267]
4.	Hemin	DIC in rats	Inhibition of TLR-5/NF-κβ/TNF-α signaling pathway	↓ MDA, TNF-α, NF-κβ, and caspase-3 levels	[268]
5.	Resveratrol	DIC in rats	Inhibition of the cardiac TLR-4 expression	↓ Troponin-I, BNP, and CK-MB levels ↓ TNF-α and IL-6 levels	[269]
6.	CTRP5	DIC in mice	Suppression of the TLR4/NLRP3 pathway	↓ Oxidative stress ↓ Inflammatory response	[270]
7.	Cilostazol	DIC in mice	Downregulation of the cardiac TLR-4 expression	↓ Levels of BNP, NF-κβ, and COX-2 expression ↓ Oxidative stress	[271]
8.	LCZ696	DIC in mice	Inhibition of the formation of TLR2-MyD88 complexes	↓ Proinflammatory markers	[272]
9.	Baicalin	DIC in rats	Inhibition of the TLR4/Iκĸ-αβ/NF-κβ pathway	↓ Inflammation ↓ Apoptosis ↓ Oxidative stress	[273]
10.	Crocin	DIC in rats	Suppression of the TLR-2/NF-κβ expression	↓ Oxidative stress ↓ Inflammatory response ↓ Calcium dysregulation	[274]
11.	Acovenoside A	DIC in mice	Inhibition of NF-κβ and IRF3/7	↓ cTnT, LDH, and CK-MB levels ↓ CRP, TNF-α, and IL-6 levels ↓ Oxidative stress	[275]
12.	Curcumin	DIC in rats	Downregulation of the level of IFN-γ	↓ NF-κβ, TNF-α, and iNOS levels ↓ cTn1 and AST levels	[276]
13.	Montelukast	DIC in rats	Suppression of the NF-κβ/TNF-α signaling pathway	↑ AREs ↓ Caspase 3	[263]
14.	Apremilast	DIC in rats	Inhibition of the NF-κβ pathway	↓ LDH and CK-MB levels ↓ ROS by upregulation of AREs ↓ Apoptosis	[264]
15.	Ellagic acid	DIC in mice	Downregulation of ERK 1/2 and JNK expressions	↑ AREs ↓ Apoptosis ↓ NF-κβ	[277]
16.	Tannic acid	DIC in rats	Inhibition of JNK expression	↓ CK-MB and LDH levels ↓ TNF-α, NF-κβ, IL-1β and caspase 3 levels ↓ NF-κβ expression	[278]
17.	Diosgenin	DIC in mice	Inhibition of the NF-κβ pathway	↑ AREs ↓ Caspase 3	[279]
18.	Irbesartan	DIC in rats	Suppression of the p38-MAPK/NF-κβ pathway	↓ ROS/RNS ↓ Caspase-3 and TGF-1β levels ↓ Inflammation	[280]
19.	Aspernolide F	DIC in rats	Suppression of the NF-κβ pathway	↓ Troponin T, LDH, and CK-MB levels ↓ TNF-α, NF-κβ, and caspase 3 levels	[281]
20.	6-gingerol	DIC in rats	Inhibition of the NF-κβ pathway	↓ TNF-α, NF-κβ and caspase 3 levels	[261]
21.	Sinapic Acid	DIC in rats	Inhibition of NF-κβ expression	↓ Troponin T, LDH, and CK-MB levels ↓ IL-1β, NF-κβ, and caspase 3 levels	[262]

**Table 6:** Pharmacological agents mitigating anthracycline-induced cardiotoxicity through inhibition of p53 pathway and NLRP3-inflammasome.

**S. No.**	**Therapeutic Agent**	**Preclinical Model**	**Molecular Mechanism**	**Outcome**	**References**
1.	Arjunolic acid	DIC in rats	Inhibition of JNK-p38 and p53 signaling pathway	↑ AREs ↓ Bcl-2 protein	[316]
2.	Pitavastatin	DIC in cultured myocytes and mice	Inhibition of p53 pathway	↓ Bcl-2 protein ↓ Oxidative stress ↓ Rac1 protein	[343]
3.	Aspalathin	DIC in H9c2 cells	Suppression of p53/mTOR/p62 signaling pathway	↓ Apoptosis	[344]
4.	Schisandrin B	DIC in mice	Inhibition of p53 pathway	↑ AREs ↓ Apoptosis and inflammation ↑ LVEF	[345]
5.	miR-146a	DIC in mice	Inhibition of the TAF9b/P53 pathway	↓ Apoptosis Regulating autophagy	[346]
6.	Resveratrol	DIC in mice	Inhibition of NLRP-3 inflammasome	↓ TNF-α, IL-6, IL-1β, and IL-18 levels	[347]
7.	Calycosin	DIC in mice	Inhibition of NLRP-3 inflammasome	↓ Oxidative stress ↑ MMP Preventing pyroptosis	[348]
8.	Fraxetin	DIC in rats	Inhibition of NLRP-3 inflammasome	↑ AREs ↑ p-MAPK, JNK activity, and Beclin-l levels	[349]
9.	Resolvin E1	DIC in endothelial cells	Inhibition of the NLRP3 inflammasome	↓ Inflammation ↓ p65, p21, and NLRP3	[350]
10.	Polyguluronic acid	DIC in C57BL/6j mice	Inhibition of NLRP-3 inflammasome	↓ LDH and IL-1β ↓ NLRP-3 and caspase-1 expression and ASC oligomerization	[351]
11.	Nicotinamide	DIC in rats	Inhibition of NLRP-3 inflammasome	↓ IL-1β and caspase 1 activity ↓ Oxidative stress ↓ Apoptosis	[352]
12.	Astragaloside IV	DIC in C57BL/6j mice	Inhibition of NLRP-3 inflammasome	↓ BNP, CK-MB, cTnI, and LDH levels ↓ Inflammation and oxidative stress	[353]

**Table 7:** List of ferroptosis inhibitors for the prevention of anthracycline-induced cardiotoxicity.

**S. No.**	**Ferroptosis Inhibitors**	***In vitro*/*In vivo* Model**	**Outcome**	**References**
1.	Aloe-emodin	Dox-induced cardiotoxicity in H9c2 rat cardiomyocytes	↓ oxidative stress; ↑Nrf2 downstream antioxidant genes (*SLC7A11*, GPX4); complexes with bivalent iron regulating the intracellular iron-related genes	[364]
2.	Chiral polymer micelles	Adriamycin-induced cardiotoxicity murine model	↓ CoQ10; ↓ lipid peroxidation; inhibited ferroptosis	[365]
3.	Ethoxyquin	DOX-induced cell death in cultured cardiomyocytes and cardiotoxicity in a murine model of Dox-induced cardiotoxicity	↓ MDA; ↓ acrolein ↓ contractile dysfunction; ↓ myocardial atrophy; ↓ lung congestion; ↓ cardiac fibrosis; inhibited ferroptosis	[366]
4.	Resveratrol	Dox-induced cardiotoxicity C57BL/6 J mice model	↑ phosphorylation levels of ERK, p38, c-JNK; ↑ GPX4; ↑ GSH; ↓ iron accumulation → improved cardiac functions	[367]
5.	WGX50 (extract derived from *Zanthoxylum bungeanum* Maxim)	DOX-induced cardiotoxicity (*in vivo* and *in vitro*)	Improved cardiac dysfunction, cardiac injury, fibrosis, mitochondrial damage, and redox imbalance; ↓ mitochondrial membrane potential; ↑ ATP production; ↓ iron accumulation; ↑ GPX4	[360]
6.	Dexrazoxane	DOX-induced cardiotoxicity rat model	↑ GPX4; ↑ FTH1; ↓ MDA	[368]

## LIST OF ABBREVIATIONS

CVDs: Cardiovascular Diseases
DIC: Doxorubicin-induced Cardiotoxicity
Dox: Doxorubicin
mTOR: Mammalian Target of Rapamycin
PFAs: Polyunsaturated Fatty Acids
ROS: Reactive Oxygen Species
TLR: Toll-like Receptor
UVRAG: Ultraviolet Radiation Resistance-associated Gene
